# *Cornus mas* and *Cornus Officinalis*—Analogies and Differences of Two Medicinal Plants Traditionally Used

**DOI:** 10.3389/fphar.2018.00894

**Published:** 2018-08-28

**Authors:** Monika E. Czerwińska, Matthias F. Melzig

**Affiliations:** ^1^Department of Pharmacognosy and Molecular Basis of Phytotherapy, Medical University of Warsaw, Warsaw, Poland; ^2^Institute of Pharmacy, Freie Universtaet Berlin, Berlin, Germany

**Keywords:** *Cornus mas*, *Cornus officinalis*, cornelian cherry, cornel dogwood, traditional Chinese medicine

## Abstract

Among 65 species belonging to the genus *Cornus* only two, *Cornus mas* L. and *Cornus officinalis* Sieb. et Zucc. (Cornaceae), have been traditionally used since ancient times. *Cornus mas* (cornelian cherry) is native to southern Europe and southwest Asia, whereas *C. officinalis* (Asiatic dogwood, cornel dogwood) is a deciduous tree distributed in eastern Asia, mainly in China, as well as Korea and Japan. Based on the different geographic distribution of the closely related species but clearly distinct taxa, the ethnopharmacological use of *C. mas* and *C. officinalis* seems to be independently originated. Many reports on the quality of *C. mas* fruits were performed due to their value as edible fruits, and few reports compared their physicochemical properties with other edible fruits. However, the detailed phytochemical profiles of *C. mas* and *C. officinalis*, in particular fruits, have never been compared. The aim of this review was highlighting the similarities and differences of phytochemicals found in fruits of *C. mas* and *C. officinalis* in relation to their biological effects as well as compare the therapeutic use of fruits from both traditional species. The fruits of *C. mas* and *C. officinalis* are characterized by the presence of secondary metabolites, in particular iridoids, anthocyanins, phenolic acids and flavonoids. However, much more not widely known iridoids, such as morroniside, as well as tannins were detected particularly in fruits of *C. officinalis*. The referred studies of biological activity of both species indicate their antidiabetic and hepatoprotective properties. Based on the available reports antihyperlipidemic and anticoagulant activity seems to be unique for extracts of *C. mas* fruits, whereas antiosteoporotic and immunomodulatory activities were assigned to preparations of *C. officinalis* fruits. In conclusion, the comparison of phytochemical composition of fruits from both species revealed a wide range of similarities as well as some constituents unique for cornelian cherry or Asiatic dogwood. Thus, these phytochemicals are considered the important factor determining the biological activity and justifying the use of *C. mas* and *C. officinalis* in the traditional European and Asiatic medicine.

## Introduction

Recently published ethnopharmacological studies regarding the genus *Cornus* (Cornaceae) have shown that among 65 species only two of them have a long tradition in a medicinal use. *Cornus mas* L. native to southern Europe and southwest Asia, known as cornelian cherry, is used since ancient times (Dragendorff, 1898). The fruits of this plant were used for a broad variety of diseases and complaints in all areas of its geographical distribution. The preparations from *C. mas* (European cornel, cornelian cherry) were considered as astringent, tonic and antipyretic remedies (Fournier, 1948). *Cornus officinalis* Sieb. et Zucc., also named Asiatic dogwood (cornel dogwood, Japanese cornel), is a deciduous tree distributed in eastern Asia, mainly in China, as well as Korea and Japan (Ma et al., 2014). It was used as a traditional Chinese medicine for more than 2,000 years to promote a healthy liver and kidney, and is a common ingredient of many TCM prescriptions (Liu X. et al., 2011).

The dogwood genus *Cornus* L. is a member of Cornales in the Asterids clade (The Angiosperm Phylogeny Group; APG IV, 2016). The dogwood genus is morphologically heterogenous, variable in inflorescences, fruits and chromosomes (Eyde, 1988; Xiang et al., 2005; Ma et al., 2017). Four major lineages of the dogwood genus *Cornus* L., such as the blue- or white-fruited group, the cornelian cherry group, the big-bracted group and the dwarf dogwood group, have been drawn (Xiang et al., 2006, 2008). However, the systematical classification of flowering plants from Cornales clade and relationships within this taxon seems to be problematic for many years (Xiang et al., 1996, 1998, 2002, 2006; Fan and Xiang, 2001; APG IV, 2016). Despite these controversies, phylogenetic analyses of this clade based on four DNA regions, such as *rbcL* and *matK* from chloroplast DNA genome as well as 26S rDNA and ITS (internal transcribed spacer) 1–5. 8S rDNA from the nuclear genome, classified both *C. mas* and *C. officinalis* in the group of cornelian cherries (Xiang et al., 1998, 2002, 2006; Fan and Xiang, 2001). In addition, the subgenera *Afrocarnia* (Harms) Wangerin, *Cornus* L. and *Sinocornus* Q.Y. Xiang were included into the group of cornelian cherries. *Cornus mas* and *C. officinalis* were classified in the subgenus *Cornus* L. (Fan and Xiang, 2001; Xiang et al., 2006). Both of them are temperate deciduous small shrubs or trees blooming before leaf development. The morphological characters, such as umbellate cymes terminal subtended by four not showy scale-like bracts, red or purple black fruits and the wall of fruit stone riddled with cavities, supported the membership of these species in the monophyletic group of cornelian cherries. All these data strongly supported the sister phylogenetic relationship of *C. mas* and *C. officinalis* (Xiang et al., 2005). The genus *Cornus* is widely distributed in the northern hemisphere. Fossil fruit stones similar to the contemporary fruit stones of *C. mas* are likely to derive from the late Miocene of Poland, as well as from Pliocene and Pleistocene of France, Germany and Netherlands. Evidence from analyses of fossils, phylogeny and biogeographic distribution support an origin and early diversification of *Cornus* likely in Europe. It is suggested that the ancestor of the living cornelian cherries spread from Europe to eastern Asia around the mid-Oligocene and the mid-Miocene. *Cornus mas* is a single representative species of cornelian cherry in Europe. On the other hand, *C. officinalis* in eastern Asia represents the Miocene migrant from Europe (Xiang et al., 2005). Based on the different geographic distribution of the closely related species but clearly distinct taxa, the ethnopharmacological use of *C. mas* and *C. officinalis* seems to be independently originated. In the traditional medicine of Europe and Middle East only fruits of *C. mas* were used and in the traditional medicine of China reports describe only preparations of fruits from *C. officinalis* for medicinal application.

Plant materials belonging to the genus *Cornus* are characterized by the presence of secondary metabolites, in particular iridoids, anthocyanins, tannins and flavonoids. Although many reports on the quality of fruits of *C. mas* were performed due to the fact of their value as edible fruits, as well as few studies compared their properties with other edible fruits used for a production of wines, liquors or jams (Tarko et al., 2014; Cosmulescu et al., 2017), the detailed phytochemical profiles of *C. mas* and *C. officinalis*, in particular fruits, have never been compared. The chemical composition of plant materials obtained from *C. mas* and *C. officinalis* can be significantly different depending on the geographic location, climatic factors, season, as well as cultivation practice. For this reason, the phytochemical composition is considered an important factor determining the biological activity and justifying the traditional use of these species.

All these facts taking together make interesting to compare the therapeutic use of both traditional species based on their chemical composition. This review particularly highlights the similarities and differences in phytochemicals found in fruits of *C. mas* and *C. officinalis* in relation to their biological effects based on reports available in this field.

## Similarities and differences in the chemical composition of fruits from *C. mas* and *C. officinalis*

### General

**Figure 1:**
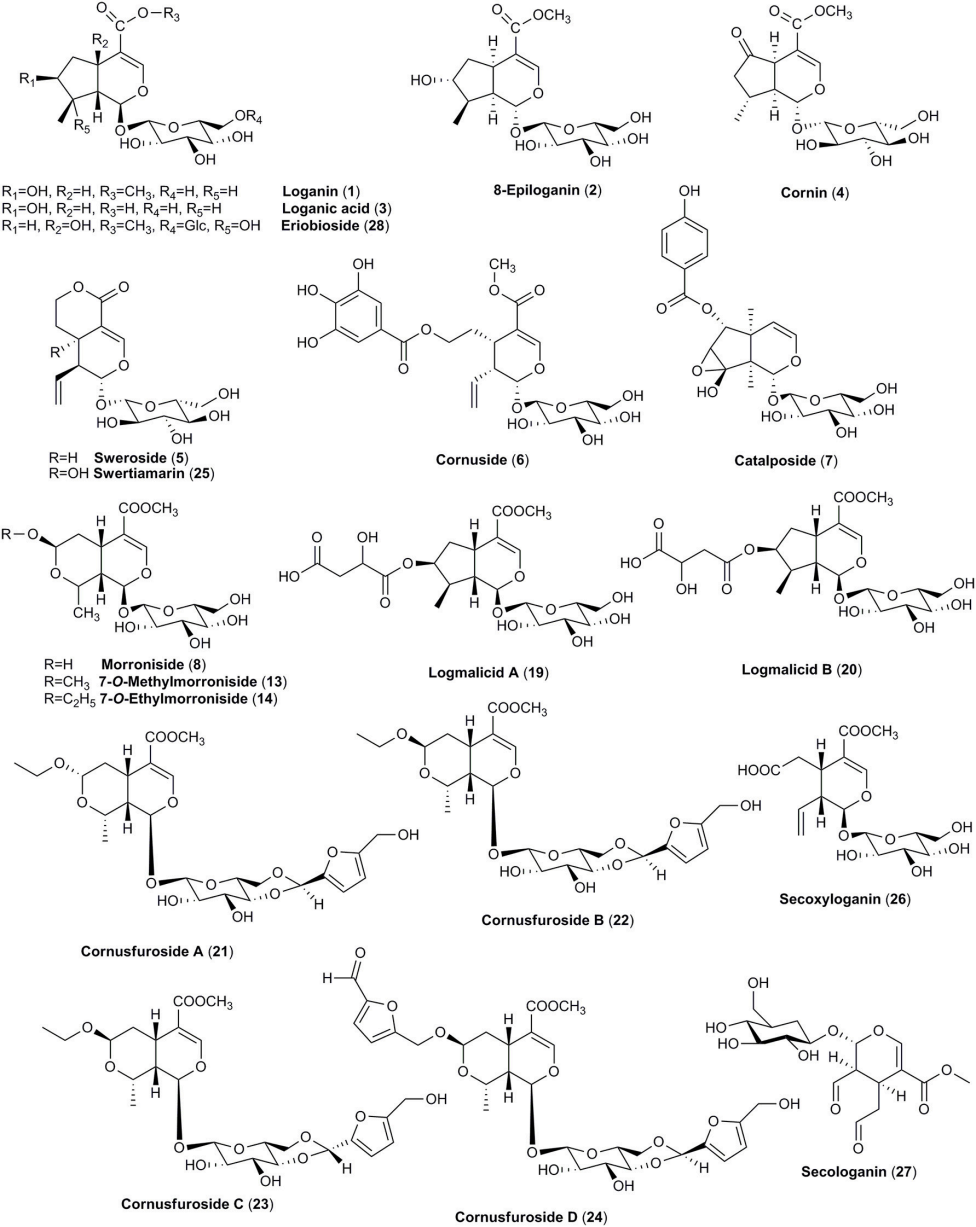
The chemical structures of iridoids identified in fruits of *C. mas* and/or *C. officinalis*.

**Figure 2:**
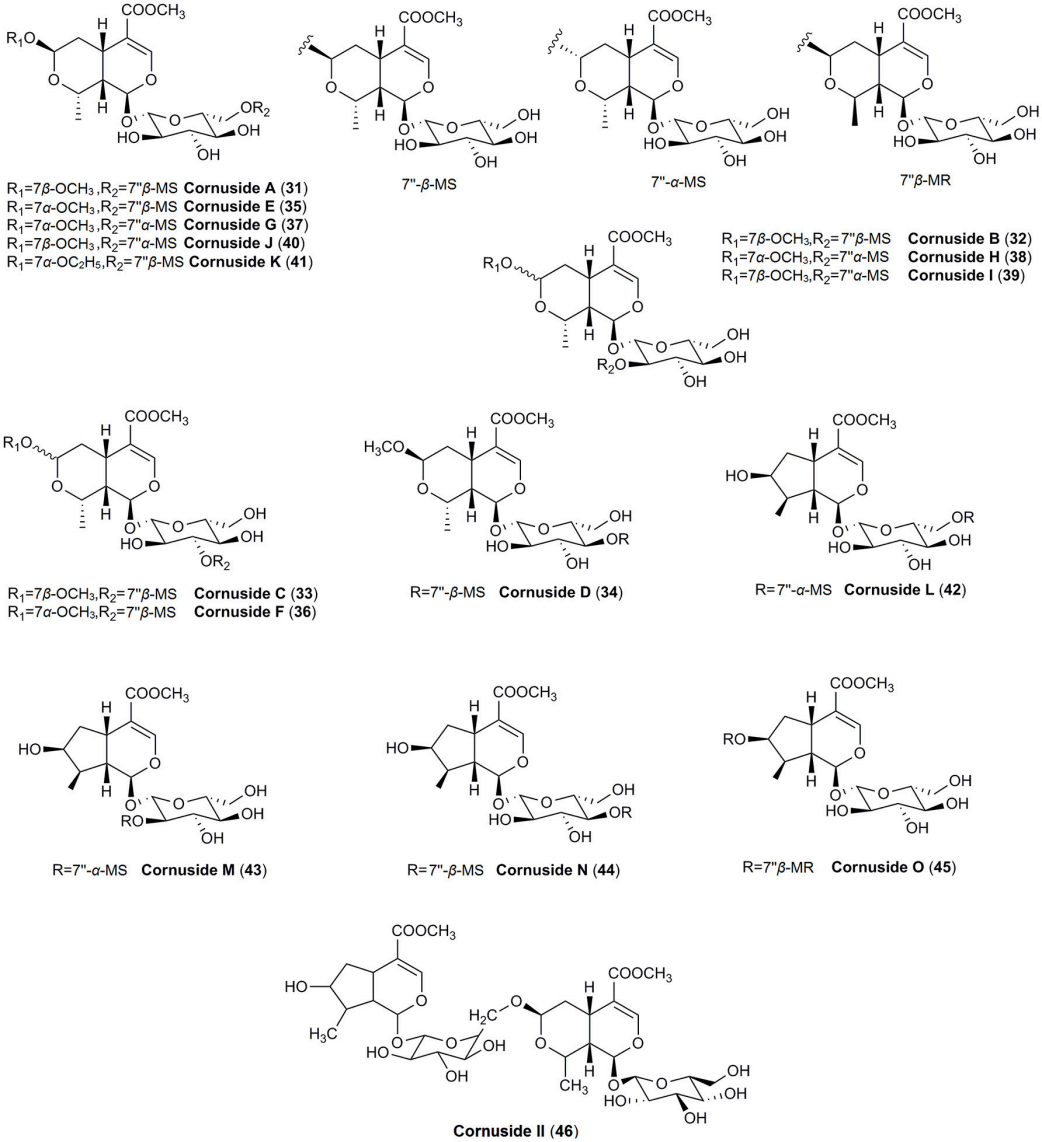
The chemical structures of iridoids identified in fruits of *C. officinalis*.

The main secondary metabolites found in dogwoods seem to be iridoid and non-flavonoid glucosides. Even though the blue-fruited dogwoods contain non-flavonoid glucoside salidroside derived from shikimic acid, iridoid glucosides were found to be unique in all red-fruited dogwoods, and they are derived from mevalonic acid (Bate-Smith et al., 1975; Jensen et al., 1975). The dogwoods iridoids were classified into three groups, including cornin and its precursor dihydrocornin, monotropein and its precursor geniposide as well as secoiridoids, secologanin and morroniside (Bate-Smith et al., 1975). Thus, the presence of iridoids (Figures 1, 2) in red-fruited dogwoods is considered a symplesiomorphy (Xiang et al., 1996), and confirms the close taxonomical relationship of *C. mas* and *C. officinalis*. The representative compounds of this group, such as loganin, loganic acid, cornin, sweroside, and cornuside, have been found in both species.

Furthermore, the polyphenolic compounds are represented by anthocyanins, flavonoids and phenolic acids in fruits of *C. mas* and *C. officinalis*. Pelargonidin 3-*O*-galactoside followed by cyanidin 3-*O*-galactoside and delphinidin 3-*O*-galactoside were the most abundant anthocyanins in fruits of both species. Among flavonoids, quercetin, and kaempferol glycosides were detected in both species. Taking into consideration that flavonoids are widely distributed in plants, their occurrence was considered the similar characteristic for both species. Nevertheless, some discrepancy in the flavonoid composition occurs. Even more flavonoids have been referred by Huang et al. (2018) but full availability of the studies concerning their identification or isolation is rather limited.

Last but not least, the similar constituents of *C. mas* and *C. officinalis* are considered triterpenoids, in particular represented by ursolic acid detected in both species, and carbohydrates. It is worth to note that neither in fruits of *C. mas* nor in fruits of *C. officinalis* phenylpropanoid esters have been reported to date, even though they usually co-exist with iridoids as it is observed in some plants from Oleaceae, Scrophulariaceae, Verbenaceae, Plantaginaceae, and Lamiaceae. This might be justified by the less significant role of shikimate pathway in biosynthesis of compounds in red-fruited dogwoods. Table 1 summarizes the key phytochemicals of fruits from *C. mas* and *C. officinalis* that can be found in the literature. The names of compounds have been provided as they were described in the referred literature. Due to the fact that HPLC-MS is currently one of the most popular method for elucidation of chemical composition of plant preparations, the molecular weights of phytochemicals were also listed (Table 1) to help reader with comparing experimental data in order to preliminary identification detected constituents.

**Table 1:** The comparison of phytochemical constituents from fruits of *C. mas* and *C. officinalis*.

**Compound no**.	**Compound name**	**Molecular weight**	***C. mas***	***C. officinalis***	**References**
**IRIDOIDS**					
1	Loganin	390	X	X	Du et al., 2008; Yokozawa et al., 2010; Cao et al., 2011, 2012; Lin et al., 2011; Lee A. Y. et al., 2012; Lee N. H. et al., 2012; Xie et al., 2012; Cai et al., 2013; Deng et al., 2013; Ma et al., 2014; Perova et al., 2014; He et al., 2016; Jiang et al., 2016; Ye et al., 2017
2	8-Epiloganin	390		X	Ye et al., 2017
3	Loganic acid	376	X	X	Yokozawa et al., 2010; Cao et al., 2011, 2012; West et al., 2012b; Deng et al., 2013; Ma et al., 2014; Sozanski et al., 2014, 2016; Kucharska et al., 2015; Antolak et al., 2017; Ji et al., 2017
4	Cornin (verbenalin)	388	X	X	Cao et al., 2011; Liu Z. et al., 2011; Cai et al., 2013; Perova et al., 2014; Jiang et al., 2016
5	Sweroside	358	X	X	Du et al., 2008; Cao et al., 2011, 2012; Liu Z. et al., 2011; Cai et al., 2013; Deng et al., 2013; Perova et al., 2014; Jiang et al., 2016; Ye et al., 2017
6	Cornuside	542	X	X	Hatano et al., 1990; Du et al., 2008; Lin et al., 2011; Liu Z. et al., 2011; Lee A. Y. et al., 2012; Deng et al., 2013; Ma et al., 2014; Perova et al., 2014; Sozanski et al., 2014; Kucharska et al., 2015; Jiang et al., 2016; Antolak et al., 2017
7	Catalposide	482	X		Sochor et al., 2014
8	Morroniside	406		X	Du et al., 2008; Yokozawa et al., 2010; Cao et al., 2011, 2012; Lin et al., 2011; Lee A. Y. et al., 2012; Lee N. H. et al., 2012; Cai et al., 2013; He et al., 2016; Jiang et al., 2016; Ji et al., 2017
9	7-α-*O*-Methylmorroniside	420		X	Ma et al., 2014; Jiang et al., 2016; Ye et al., 2017
10	7-β-*O*-Methylmorroniside	420		X	Xie et al., 2012; Ma et al., 2014; Jiang et al., 2016; Ye et al., 2017
11	7-α-*O*-Ethylmorroniside	434		X	Cao et al., 2012; Ma et al., 2014; Jiang et al., 2016; Ye et al., 2017
12	7-β-*O*-Ethylmorroniside	434		X	Cao et al., 2012; Ma et al., 2014; Jiang et al., 2016; Ye et al., 2017
13	7-*O*-Methylmorroniside	420		X	Liu Z. et al., 2011; Cai et al., 2013; Ji et al., 2017
14	7-*O*-Ethylmorroniside	434		X	Cao et al., 2011; Ji et al., 2017
15	7-β-*O*-Dimethyl butanedioate morroniside	550		X	Park et al., 2016
16	(7*R*)-*n* butyl morroniside	462		X	Lin et al., 2011
17	7-α-Morroniside	406		X	Xie et al., 2012; Ma et al., 2014; Ye et al., 2017
18	7-β-Morroniside	406		X	Xie et al., 2012; Ma et al., 2014; Ye et al., 2017
19	Logmalicid A	506		X	Ma et al., 2014
20	Logmalicid B	506		X	Ma et al., 2014
21	Cornusfuroside A	542		X	He et al., 2017
22	Cornusfuroside B	542		X	He et al., 2017
23	Cornusfuroside C	542		X	He et al., 2017
24	Cornusfuroside D	622		X	He et al., 2017
25	Swertiamarin	374		X	Cao et al., 2011; Liu Z. et al., 2011
26	Secoxyloganin	404		X	Ma et al., 2014
27	Secologanin (loniceroside)	388		X	Jensen et al., 1973
28	Eriobioside	568		X	Liu Z. et al., 2011
29	7-Dehymorroniside	404		X	Cao et al., 2012
30	7-*O*-methyloganic acid	390		X	Cao et al., 2012
31	Cornuside A	809		X	Ye et al., 2017
32	Cornuside B	809		X	Ye et al., 2017
33	Cornuside C	809		X	Ye et al., 2017
34	Cornuside D	809		X	Ye et al., 2017
35	Cornuside E	809		X	Ye et al., 2017
36	Cornuside F	809		X	Ye et al., 2017
37	Cornuside G	809		X	Ye et al., 2017
38	Cornuside H	809		X	Ye et al., 2017
39	Cornuside I	809		X	Ye et al., 2017
40	Cornuside J	809		X	Ye et al., 2017
41	Cornuside K	823		X	Ye et al., 2017
42	Cornuside L	779		X	Ye et al., 2017
43	Cornuside M	779		X	Ye et al., 2017
44	Cornuside N	779		X	Ye et al., 2017
45	Cornuside O	779		X	Ye et al., 2017
46	Cornuside II	779		X	Liu Z. et al., 2011
47	Cornuside III	506		X	Wang et al., 2017
48	Cornuside IV	506		X	Wang et al., 2017
49	Corniside I	779		X	Cao et al., 2012
50	Corniside II	542		X	Cao et al., 2011, 2012
51	Williamsoside D	822		X	Ye et al., 2017
**ANTHOCYANINS**					
52	Pelargonidin	271	X		Sozanski et al., 2016
53	Pelargonidin 3-*O*-galactoside	433	X	X	Du and Francis, 1973; Seeram et al., 2002; Jayaprakasam et al., 2006; Vareed et al., 2006; Sozanski et al., 2014, 2016; Kucharska et al., 2015
54	Pelargonidin 3-*O*-glucoside	433	X		Jayaprakasam et al., 2005; Tural and Koca, 2008; Pawlowska et al., 2010; Capanoglu et al., 2011; Milenković-Andjelković et al., 2015; Antolak et al., 2017
55	Pelargonidin 3-*O*-robinobioside (pelargonidin 3-*O*-rhamnogalactoside)	579	X		Du and Francis, 1973; Sozanski et al., 2014, 2016; Kucharska et al., 2015; Antolak et al., 2017
56	Pelargonidin 3-*O*-rutinoside	579	X		Pawlowska et al., 2010
57	Cyanidin	287	X		Sozanski et al., 2016
58	Cyanidin 3-*O*-galactoside	449	X	X	Du and Francis, 1973; Seeram et al., 2002; Jayaprakasam et al., 2005, 2006; Vareed et al., 2006; Pawlowska et al., 2010; Begic-Akagic et al., 2013; Drkenda et al., 2014; Sozanski et al., 2014, 2016; Kucharska et al., 2015; Milenković-Andjelković et al., 2015
59	Cyanidin 3-*O*-glucoside	449	X		Tural and Koca, 2008; Capanoglu et al., 2011; Antolak et al., 2017
60	Cyanidin 3-*O*-robinobioside (cyanidin 3-*O*-rhamnogalactoside)	595	X		Du and Francis, 1973; Sozanski et al., 2014, 2016; Kucharska et al., 2015; Antolak et al., 2017
61	Cyanidin 3-*O*-rutinoside	595	X		Du and Francis, 1973; Tural and Koca, 2008; Capanoglu et al., 2011; Begic-Akagic et al., 2013; Drkenda et al., 2014
62	Peonidin 3-*O*-glucoside	464	X		Begic-Akagic et al., 2013; Drkenda et al., 2014
63	Delphinidin 3-*O*-glucoside	465		X	Jayaprakasam et al., 2005
64	Delphinidin 3-*O*-galactoside	465	X	X	Du and Francis, 1973; Seeram et al., 2002; Jayaprakasam et al., 2006; Vareed et al., 2006; Sozanski et al., 2014, 2016; Kucharska et al., 2015; Milenković-Andjelković et al., 2015
65	Petunidin 3-glucoside	479	X		Antolak et al., 2017
**FLAVONOIDS**					
**Flavonols**					
66	Quercetin	302	X	X	Kim and Kwak, 1998; Xie et al., 2012; Radovanović et al., 2013; Drkenda et al., 2014; Ma et al., 2014; Sochor et al., 2014; Antolak et al., 2017; Cosmulescu et al., 2017
67	Quercetin 3-*O*-β-D-xyloside (avicularin)	434	X		Pawlowska et al., 2010; Begic-Akagic et al., 2013; Drkenda et al., 2014
68	Quercetin 3-*O*-α-L-rhamnoside (quercitrin)	448	X		Pawlowska et al., 2010; Begic-Akagic et al., 2013; Drkenda et al., 2014; Sochor et al., 2014; Popović et al., 2017; Bajić-Ljubičić et al., 2018
69	Quercetin 3-*O*-β-D-glucoside (isoquercetin, isoquercitrin)	464	X	X	Pawlowska et al., 2010; Liu Z. et al., 2011; Xie et al., 2012; Begic-Akagic et al., 2013; Drkenda et al., 2014; Ma et al., 2014; Milenković-Andjelković et al., 2015; Antolak et al., 2017; Popović et al., 2017; Bajić-Ljubičić et al., 2018
70	Quercetin 3-*O*-β-D-galactoside (hyperoside)	464	X	X	Pawlowska et al., 2010; Xie et al., 2012; Begic-Akagic et al., 2013; Drkenda et al., 2014; Ma et al., 2014; Milenković-Andjelković et al., 2015; Popović et al., 2017; Bajić-Ljubičić et al., 2018
71	Quercetin 3-*O*-β-D-glucuronide (querciturone)	478	X	X	Pawlowska et al., 2010; Lin et al., 2011; Liu Z. et al., 2011; Popović et al., 2017; Bajić-Ljubičić et al., 2018
72	Quercetin 3-*O*-rutinoside (rutin)	610	X		Pawlowska et al., 2010; Begic-Akagic et al., 2013; Deng et al., 2013; Radovanović et al., 2013; Drkenda et al., 2014; Sochor et al., 2014; Milenković-Andjelković et al., 2015; Rudrapaul et al., 2015; Cosmulescu et al., 2017; Popović et al., 2017; Bajić-Ljubičić et al., 2018
73	Quercetin 3-*O*-robinobioside	610	X		Begic-Akagic et al., 2013; Drkenda et al., 2014
74	Quercetin 3-*O*-β-D-glucuronide methyl ester	492		X	Ma et al., 2014
75	Quercetin 3-*O-β*-D-(6″-*n*-butyl glucuronide)	534		X	Lin et al., 2011
76	Kaempferol	286		X	Kim and Kwak, 1998; Xie et al., 2012
77	Kaempferol 3-*O*-β-D-galactoside (trifolin)	448	X	X	Pawlowska et al., 2010; Ma et al., 2014
78	Kaempferol 3-glucoside	448	X		Begic-Akagic et al., 2013; Drkenda et al., 2014; Milenković-Andjelković et al., 2015
79	Kaempferol 3-*O*-β-D-rutinoside	594		X	Ma et al., 2014
80	4′-Methylkaempferol (kaempferide)	300		X	Xie et al., 2012
81	Myricetin	318	X		Rudrapaul et al., 2015; Cosmulescu et al., 2017
82	Myricetin 3-galactoside	480	X		Antolak et al., 2017
83	2*R*, 3*R*-*trans*-Aromadendrin	288	X		Rudrapaul et al., 2015
84	Aromadendrin 7-*O*-β-D-glucoside (sinensin)	450	X		Pawlowska et al., 2010
85	Naringenin 7-*O*-methylether	286	X		Rudrapaul et al., 2015
86	7,3′-Dihydroxy-5,4′-dimethoxyflavanone	316	X		Rudrapaul et al., 2015
87	4-Acetoxy-5,2′,4′,6′-β-pentahydroxy-3-methoxychalcone	376	X		Rudrapaul et al., 2015
**Flavan-3-ols**					
88	(+) Catechin	290	X		Radovanović et al., 2013; Milenković-Andjelković et al., 2015
89	(-) Epicatechin	290	X		Radovanović et al., 2013; Milenković-Andjelković et al., 2015; Cosmulescu et al., 2017
90	(-) Epicatechin-3-*O*-gallate	442		X	Lin et al., 2011
91	Procyanidin B1	578	X		Begic-Akagic et al., 2013; Drkenda et al., 2014
92	Procyanidin B2	578	X		Begic-Akagic et al., 2013; Radovanović et al., 2013; Drkenda et al., 2014; Milenković-Andjelković et al., 2015
**PHENOLIC ACIDS**					
93	Gallic acid	170	X	X	Tian et al., 2000; Du et al., 2008; Cao et al., 2011; Lin et al., 2011; Liu Z. et al., 2011; Cao et al., 2012; Youn and Jun, 2012; Cai et al., 2013; Deng et al., 2013; Radovanović et al., 2013; Sochor et al., 2014; Milenković-Andjelković et al., 2015; Rudrapaul et al., 2015; Jiang et al., 2016; Antolak et al., 2017; Cosmulescu et al., 2017
94	Caffeic acid	180	X	X	Lin et al., 2011; Radovanović et al., 2013; Ma et al., 2014; Cosmulescu et al., 2017
95	*p*-Coumaric acid (coumaric acid)	164	X	X	Youn and Jun, 2012; Radovanović et al., 2013; Antolak et al., 2017; Cosmulescu et al., 2017
96	Ellagic acid	302	X	X	Cao et al., 2012; Milenković-Andjelković et al., 2015; Cosmulescu et al., 2017
97	Protocatechuic acid	154	X	X	Liu Z. et al., 2011; Antolak et al., 2017
98	Benzoic acid	122	X		Krivoruchko, 2014
99	Cinnamic acid (*trans*-cinnamic acid)	148	X		Antolak et al., 2017; Cosmulescu et al., 2017
100	Ferulic acid	194	X		Krivoruchko, 2014; Antolak et al., 2017; Cosmulescu et al., 2017
101	Sinapic acid	224	X		Cosmulescu et al., 2017
102	Salicylic acid	138	X		Krivoruchko, 2014; Cosmulescu et al., 2017
103	Syringic acid	198	X		Radovanović et al., 2013; Cosmulescu et al., 2017
104	Vanillic acid	168	X		Krivoruchko, 2014; Cosmulescu et al., 2017
105	Rosmarinic acid	360	X		Antolak et al., 2017
106	Chlorogenic acid	354	X		Begic-Akagic et al., 2013; Deng et al., 2013; Drkenda et al., 2014; Sochor et al., 2014; Milenković-Andjelković et al., 2015; Cosmulescu et al., 2017
107	Neochlorogenic acid	354	X		Popović et al., 2017; Bajić-Ljubičić et al., 2018
108	Tachioside	302		X	Ma et al., 2014
109	3,5-Dihydroxy-2-(2-methoxy-2-oxoethyl) phenyl 4-hydroxybenzoate	318		X	Ma et al., 2014
110	Caffeoyltartaric acid dimethyl ester	340		X	Park et al., 2016
111	Caftaric acid monomethyl ester	326		X	Lin et al., 2011
**TRITERPENOIDS**					
112	Ursolic acid	456	X	X	Jayaprakasam et al., 2006; Wang H. et al., 2008; Yu et al., 2009; Youn and Jun, 2012; He et al., 2016
113	Oleanolic acid	456		X	Wang H. et al., 2008; Lin et al., 2011
114	Arjunglucoside I	666		X	Liu Z. et al., 2011
115	Arjunglucoside II	650		X	Liu Z. et al., 2011
**CARBOHYDRATES**					
116	Calcium pectate	–	X		Bilejić et al., 2011; Jaćimović et al., 2015
117	FCAP1	–		X	Yang et al., 2010
118	PFCC-I	75,700		X	Li et al., 2012
119	PFCA-III	17,400		X	Li et al., 2012
**TANNINS**					
120	7-*O*-galloyl-D-sedoheptulose	362		X	Yamabe et al., 2009; Yokozawa et al., 2010; Liu Z. et al., 2011; Park et al., 2015
121	2,3-Di-*O*-galloyl-β-D-glucose	484		X	Okuda et al., 1984; Hatano et al., 1989a; Cao et al., 2011, 2012
122	1,7-Di-*O*-galloyl-D-glucose	484		X	Liu Z. et al., 2011
123	1,2,3-Tri-*O*-galloyl-β-D-glucose	636		X	Hatano et al., 1989a; Cao et al., 2011, 2012; Bhakta et al., 2017
124	1,2,6-Tri-*O*-galloyl-β-D-glucose	636		X	Okuda et al., 1984; Hatano et al., 1989a
125	1,2,3,6-Tetra-*O*-galloyl-β-D-glucose	788		X	Okuda et al., 1984; Hatano et al., 1989a, 1990; Cao et al., 2011, 2012; Bhakta et al., 2017
126	Tellimagrandin I	786		X	Okuda et al., 1984; Hatano et al., 1989a; Cao et al., 2012; Bhakta et al., 2017
127	Tellimagrandin II (tellimagrandin-α, eugeniin)	938		X	Okuda et al., 1984; Hatano et al., 1989a, 1990; Cao et al., 2011, 2012; Bhakta et al., 2017
128	Cornusiin A	1,571		X	Okuda et al., 1984; Hatano et al., 1989a
129	Cornusiin B	1,087		X	Okuda et al., 1984; Hatano et al., 1989a
130	Cornusiin C	2,572		X	Hatano et al., 1989a
131	Cornusiin D	1,722		X	Hatano et al., 1989b
132	Cornusiin E	1,875		X	Hatano et al., 1989b
133	Cornusiin F	2,202		X	Hatano et al., 1989b
134	Cornusiin G	1,725		X	Hatano et al., 1990
135	Camptothin A	1,419		X	Hatano et al., 1989b
136	Camptothin B	1,723		X	Hatano et al., 1989b
137	Gemin D (3-*O*-galloyl-4,6-(S)-hexahydroxydiphenoyl-D-glucose)	634		X	Okuda et al., 1984; Hatano et al., 1989a
138	Oenothein C	784		X	Okuda et al., 1984; Hatano et al., 1989a
139	Isoterchebin	955		X	Okuda et al., 1981, 1984; Hatano et al., 1989a; Bhakta et al., 2017
**CAROTENOIDS**					
140	(13*Z*) + (13′*Z*)-Lutein	568	X		Horváth et al., 2007
141	(9*Z*) + (9′*Z*)-Lutein	568	X		Horváth et al., 2007
142	(9′*Z)*-Neoxanthin	601	X		Horváth et al., 2007
143	*(E)*-Neoxanthin	601	X		Horváth et al., 2007
144	Lutein-5,6-epoxide	585	X		Horváth et al., 2007
145	Luteoxanthin (epimers)	601	X		Horváth et al., 2007
146	Neochrome (epimers)	553	X		Horváth et al., 2007
147	β-carotene	537	X		Horváth et al., 2007
148	β-carotene-5,6-monoepoxide	553	X		Horváth et al., 2007
149	β-cryptoxanthin	553	X		Horváth et al., 2007
**FATTY ACIDS**					
150	Behenic acid	340	X		Krivoruchko, 2014
151	Lauric acid	200	X		Brindza, 2009; Krivoruchko, 2014
152	Linoleic acid	280	X		Brindza, 2009; Krivoruchko, 2014
153	Linolenic acid	278	X		Brindza, 2009; Krivoruchko, 2014
154	Myristic acid	228	X		Brindza, 2009
155	Oleic acid	282	X		Brindza, 2009; Krivoruchko, 2014
156	Palmitic acid	256	X		Brindza, 2009; Krivoruchko, 2014
157	Plamitoleic acid	254	X		Brindza, 2009; Krivoruchko, 2014
158	Pentadecenic acid	240	X		Brindza, 2009
159	Pentadecanoic acid	242	X		Krivoruchko, 2014
160	Stearic acid	284	X		Brindza, 2009; Krivoruchko, 2014
161	Vaccenic acid	282	X		Brindza, 2009
162	*cis*-10,12-Octadecadienic acid	280	X		Brindza, 2009
**OTHER CONSTITUENTS**					
163	5-Hydroxymethylfurfural (5-Hydroxymethyl-2-furaldehyde)	126		X	Du et al., 2008; Yokozawa et al., 2010; Cao et al., 2011, 2012; Cai et al., 2013; Jiang et al., 2016
164	Dimethyltetrahydrofuran *cis*-2,5-dicarboxylate	188		X	Kim and Kwak, 1998
165	β-sitosterol	415		X	Lin et al., 2011
166	Daucosterol-6′-malate	692		X	Xie et al., 2012
167	(1′*S*, 2′*R*)-guaiacyl glycerol 3′-O-β-D-glucoside	376		X	Ma et al., 2014
168	Mevaloside	292		X	Yokozawa et al., 2010
169	2,7-Anhydro-D-sedoheptulose (sedoheptulosan)	192		X	Liu Z. et al., 2011
170	Resveratrol	228	X		Sochor et al., 2014
171	Malic acid	134	X	X	Cao et al., 2012; Begic-Akagic et al., 2013; Deng et al., 2013; Drkenda et al., 2014; Krivoruchko, 2014
172	Malic acid methyl ester	148		X	Cao et al., 2011, 2012
173	Dimethyl malate	162		X	Miyazawa et al., 2003
174	Tartaric acid	150	X	X	Cao et al., 2012; Begic-Akagic et al., 2013; Deng et al., 2013; Drkenda et al., 2014
175	Butoxysuccinic acid	190		X	Lin et al., 2011
176	Citric acid	192	X		Begic-Akagic et al., 2013; Deng et al., 2013; Drkenda et al., 2014
177	Succinic acid	118		X	Zhang Q. C. et al., 2013

### Similarities of chemical composition

#### Iridoids

It is believed that particularly loganin (**1**) plays a crucial role in the formation sequences of highly oxidized glucosides of iridoids as well as in the biosynthesis of secoiridoids glucosides and indole alkaloids. The cyclization to a hemiacetal is likely to take place through the incorporation of an oxygen function into the C-8 position of secologanin (**27**) derived from loganin (**1**) to generate further iridoid derivatives (Inouye et al., 1974). Secoiridoids are believed to be even more advanced due to the more complicated biostynthetic steps (Xiang et al., 1996). Thus, the most closely related in the iridoids transformation pathway compounds, such as loganin (**1**), loganic acid (**3**), cornin (**4**), sweroside (**5**), and representative of secoiridoids, cornuside (**6**), have been detected both in fruits of *C. mas* and *C. officinalis* (Deng et al., 2013; Ma et al., 2014; Perova et al., 2014; Sochor et al., 2014; Kucharska et al., 2015; Jiang et al., 2016). However, it should be noted that in the 26 cultivars of *C. mas* it was shown that the most abundant iridoid was loganic acid (**3**) (81.5–461.1 mg/100 g fw) constituting 88–96% of total iridoids (86.9–493.7 mg/100 g fw). Cornuside (**6**) has already followed loganic acid (**3**) but its content was much lower than loganic acid (**3**) and maximally reached 36.2 mg/100 g fw (West et al., 2012a; Kucharska et al., 2015). Only two reports describe the identification of loganin (**1**) and sweroside (**5**) in fruit of *C. mas* (Deng et al., 2013; Perova et al., 2014). Nevertheless, the reason of limited data on identification of loganin and sweroside in fruits of cornelian cherry is due to their low concentration compared to loganic acid. More detailed studies are needed in this field.

#### Anthocyanins

**Figure 3:**
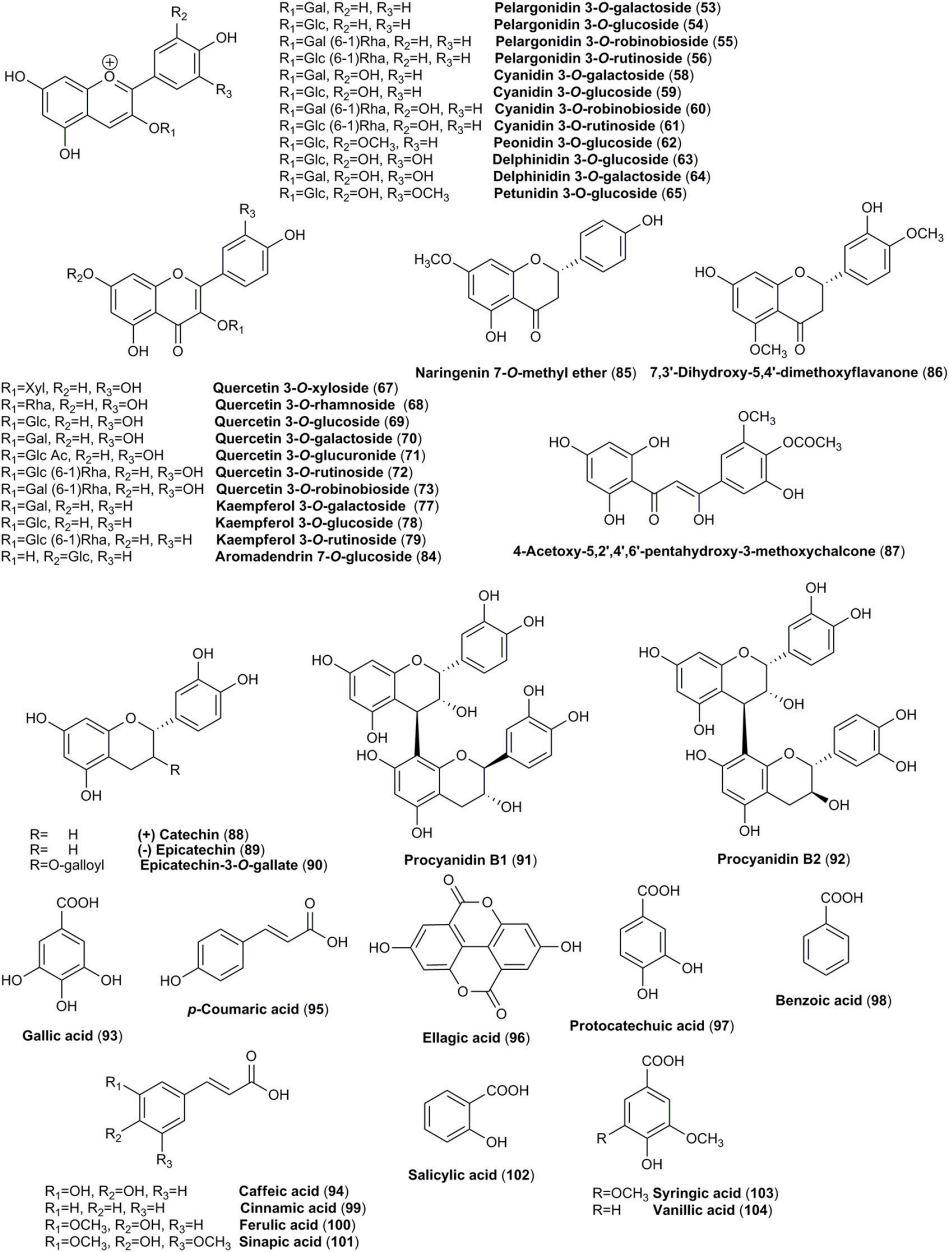
The chemical structures of anthocyanins, flavonoids, and phenolic acids identified in fruits of *C. mas* and/or *C. officinalis*.

The mature fruits from both *C. mas* and *C. officinalis* are dark red colored and for this reason they might be easily confused. Thus, in addition to iridoids (Figures 1, 2), anthocyanins (Figure 3), red colorant compounds, are widely distributed in fruits of these dogwoods. The content of anthocyanins increases when the fruits change their color from light yellow to dark red (Gunduz et al., 2013). Fruits of cornelian cherry and Asiatic dogwood contain in particular pelargonidin (**52**) and cyanidin (**57**) derivatives. Both in *C. mas* and *C. officinalis* pelargonidin 3-*O*-galactoside (**53**), cyanidin 3-*O*-galactoside (**58**), and delphinidin 3-*O*-galactoside (**64**) were detected and quantified. Pelargonidin 3-*O*-galactoside (**53**) (1.62 mg/g) and cyanidin 3-*O*-galactoside (**58**) (1.66 mg/g) have been noted as the most abundant anthocyanins of *C. mas* fresh fruit (Jayaprakasam et al., 2006; Vareed et al., 2006; Sozanski et al., 2014; Kucharska et al., 2015). The amount of pelargonidin 3-*O*-galactoside (**53**) in fruit of *C. officinalis* (0.78 mg/g) was approximately half of the concentration determined in fruit of *C. mas*. The other detected anthocyanins of *C. officinalis* fruit were present even at lower concentrations (0.15–0.21 mg/g; Vareed et al., 2006). In the literature, more attention has been focused on anthocyanins of *C. mas* (Pantelidis et al., 2007; Popović et al., 2012; Bilejić et al., 2015; Kucharska et al., 2015). These pigments were identified using methods such as HPLC, LC-ES/MS and NMR (Seeram et al., 2002; Kucharska et al., 2015), HPLC (Tural and Koca, 2008; Capanoglu et al., 2011) and LC-PDA-MS (Pawlowska et al., 2010). In some cultivars of the southeastern Europe peonidin 3-*O*-glucoside (**62**) followed by cyanidin 3-*O*-galactoside (**58**) was established as the major constituent of *C. mas* fruits (Drkenda et al., 2014).

#### Flavonoids

*Cornus* species have been proven to contain flavonols mostly quercetin (**66**) and kaempferol (**76**) derivatives (Figure 3). Most of reported compounds were identified as monoglycosides containing glucose, galactose, rhamnose, and glucuronic acid as a sugar moiety. The quercetin glycosides (**69–71**) as well as kaempferol 3-*O*-β-D-galactoside **(77)** were determined in both species (Pawlowska et al., 2010; Xie et al., 2012; Ma et al., 2014). The trace amounts of aglycone of flavonols such as quercetin (**66**) was found in fruits of both *C. mas* and *C. officinalis* (Xie et al., 2012; Rudrapaul et al., 2015).

#### Phenolic acids

Three different groups of phenolic acids (Figure 3), including benzoic acid, *trans*-cinnamic acid and ellagic acid derivatives have been described in the fruits of *C. mas* and *C. officinalis*. The typical representatives of each group such as gallic acid (**93**), caffeic acid (**94**), *p*-coumaric acid (**95**), ellagic acid (**96**), and protocatechuic acid (**97**) have been found in both dogwoods (Liu Z. et al., 2011; Cao et al., 2012; Radovanović et al., 2013; Milenković-Andjelković et al., 2015; Rudrapaul et al., 2015; Antolak et al., 2017; Cosmulescu et al., 2017). Ellagic acid (**96**), which is a marker of ellagitannins occurrence, detected in the fruits of cornelian cherry suggests the presence of ellagitannins in this plant material. However, this hypothesis requires further investigation.

#### Triterpenoids

**Figure 4:**
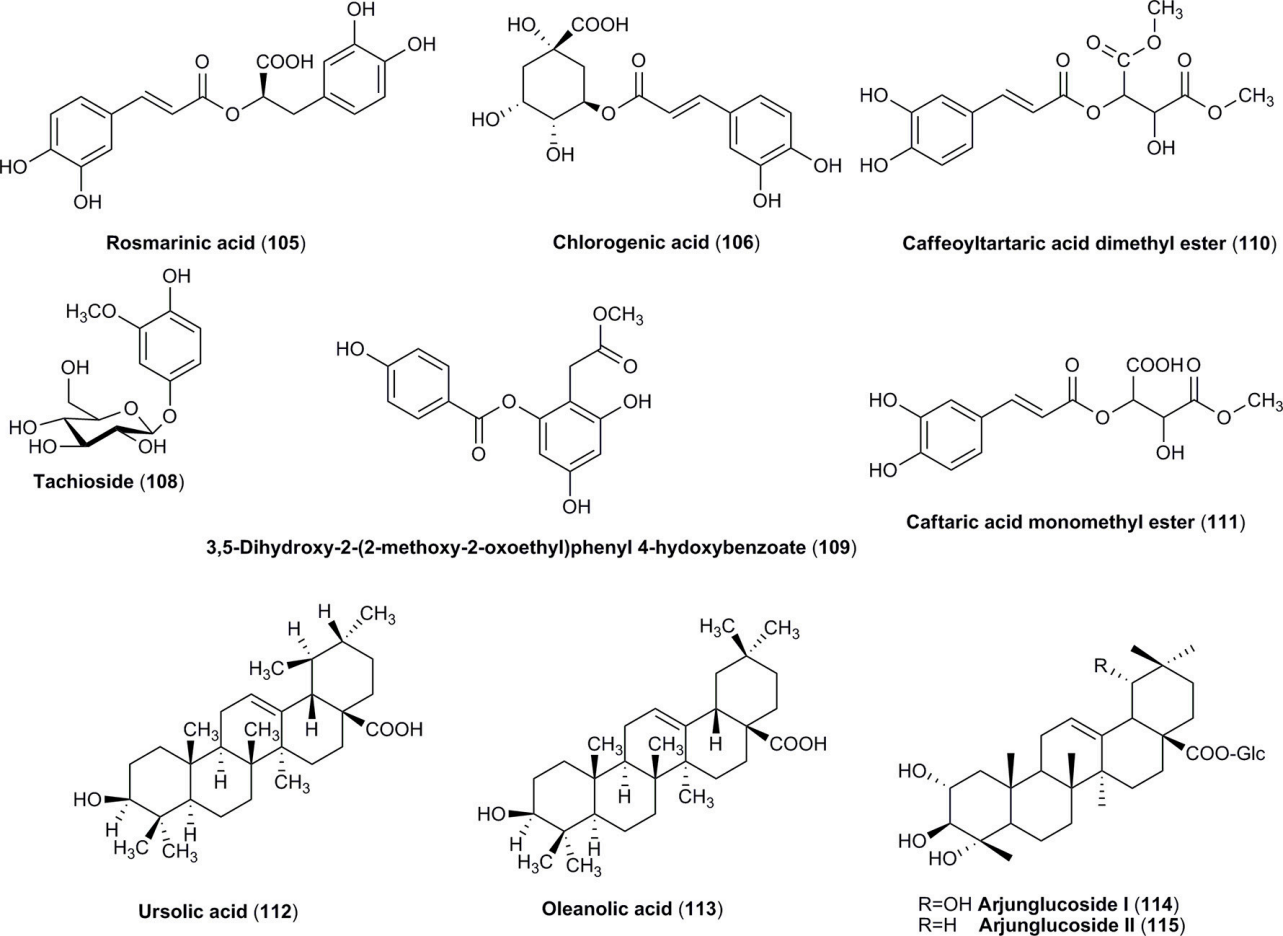
The chemical structures of phenolic compounds and terpenoids identified in fruits of *C. mas* and/or *C. officinalis*.

Additionally, some other compounds such as triterpenoids (Figure 4) and carbohydrates have been identified in both species. Among terpenoids only ursolic acid (**112**) has been reported in the fruits of cornelian cherry and Asiatic dogwood (Jayaprakasam et al., 2006; Wang H. et al., 2008; Jang et al., 2014; He et al., 2016). This compound was often reported as an active constituent found in ethanolic and methanolic extracts from fruits of *C. officinalis* (Gao et al., 2008). In addition, other three triterpenoid acids (**113–115**), including oleanolic acid, have been identified in *C. officinalis* (Jayaprakasam et al., 2006; Wang H. et al., 2008; Jang et al., 2014; He et al., 2016).

#### Carbohydrates

Carbohydrates are the crucial compounds determining the quality of fruits due to the fact that the sugar content is a major parameter affecting a direct consumption of fruits in addition to food preservation aspects (Çopur et al., 2003; Brindza, 2007; Bilejić et al., 2011, 2015). Thus, monosaccharides and polysaccharides are present in the fruits of both species. The fruits of *C. mas* contain the atypical fibers such as calcium pectate (**116**) (1.31%; Bilejić et al., 2011; Jaćimović et al., 2015). The crude fiber (0.36–0.76%) and total pectin (0.26–1.04%) contents were evaluated in *C. mas* fruits grown in different regions of Europe (Çopur et al., 2003; Sochor et al., 2014). Therefore, the juice of *C. mas* is considered an alternative fermentation substrate for the production of functional beverage. The simultaneous decreasing total sugars content and increasing contents of lactic and acetic acids were studied in the juice as well as unripe cornelian cherries fruits fermented by *Lactobacillus* sp. (Nouska et al., 2016; Czyzowska et al., 2017). It is worth to note that fermented cornelian cherry-derived products of iridoid glycosides and phenolic acids may complement gut microbiota and in this manner play a role of new probiotic functional products (Czyzowska et al., 2017). Additionally, the fibers and particularly oligosaccharides, which are generally found in fruits, are considered the non-digestible food ingredient stimulating the growth or selective activity of gut microbiota (Gibson, 2008). The dietary fiber in both cornelian cherries (Nawirska-Olszanska et al., 2010) and cornel dogwood fruits (Shao, 2011) is the important constituent of a diet determining human well-being due to a wide range of metabolic health benefits such as reduction of cholesterol level, blood pressure and body weight. Thus, the potential prebiotic activity of constituents such as dietary fiber from fruits of both dogwoods should not be excluded. On the other hand, a purification and isolation of bioactive polysaccharides of *C. officinalis* fruit (Yang et al., 2010; Li et al., 2012; You et al., 2013; Yin et al., 2016) have been also shown. The list of polysaccharides (**117–119**) identified in the fruits of *C. officinalis* is presented in Table 1. The most abundant monosaccharide components of these polysaccharides were glucose, rhamnose and arabinose (Yang et al., 2010; Li et al., 2012). The monosaccharide profile of *C. mas* fruits have been represented by glucose, fructose and sucrose (Bilejić et al., 2011; Perova et al., 2014; Tarko et al., 2014; Antolak et al., 2017).

### Differences in chemical composition

#### General

Despite a wide range of similarities and close phylogenetic relationship of *C. mas* and *C. officinalis*, few differences in chemical composition of fruits can be found. The most valuable characteristic established only in fruits of *C. officinalis* seems to be the presence of tannins (**120–139**) as well as unique iridoids. Morroniside (**8**) is one of the most important iriodid glucoside reported in the fruits of *C. officinalis*. Additionally, 43 other iridoids (**9–51**) have been identified in this plant material. It should be underlined that co-existence of iridoids and tannins seems to be very rare in plants. Furthermore, 5-hydroxymethylfurfural (**163**) and steroids, such as β-sitosterol (**165**) and daucosterol-6′-malate (**166**) are unique for *C. officinalis*.

On the other hand, flavan-3-ols derivatives, such as procyanidin B1 (**91**) and B2 (**92**), carotenoids (**140–149**) and fatty acids (**150–162**) have been identified only in fruits of *C. mas*. It is worth to note that many reports provide data on ascorbic acid or pectin content in fruits of *C. mas* due to their dietary role. As far as the Asiatic dogwood is concerned, these data are completely missed.

The one comparative study on quantities of secondary metabolites in aqueous extracts from leaves of *C. mas* and *C. officinalis* have revealed that extracts of *C. mas* are characterized by lower content of flavonoids, hydroxycinnamic acids, total polyphenols and tannins than extracts of *C. officinalis* (Forman et al., 2015). On the other hand, no comparative quantitation of major secondary metabolites, except for anthocyanins (Seeram et al., 2002; Vareed et al., 2006) of dogwood fruits is available to date. Thus, further comparative elucidation of chemical composition as well as quantification of phytochemicals in fruits of both species still seem to be a challenge.

#### Unique iridoids of *C. officinalis*

Although iridoids were classified as similar characteristic for both species, much more not widely known iridoids have been found only in fruits of *C. officinalis*. In particular, in contrary to the cornelian cherry fruits the major iridoid constituents of Asiatic dogwood fruits were isomers of α and β of morroniside (**8**) as well as their methyl and ethyl derivatives (**9–15**) (Du et al., 2008; West et al., 2012a,b; Xie et al., 2012; Jiang et al., 2016; Park et al., 2016; Ji et al., 2017). Additionally, apart from typical iridoids described previously, less-known compounds such as logmalicids A and B (**19–20**) as well as cornusfurosides A-D (**22–24**) were found in fruits of *C. officinalis* (Ma et al., 2014; He et al., 2017). Fifteen rare iridoid glucoside dimers, named cornusides A-O (**31–45**) (Figure 2), have been isolated from an aqueous extract of *C. officinalis* fruits (Ye et al., 2017).

#### Tannins of *C. officinalis*

**Figure 5:**
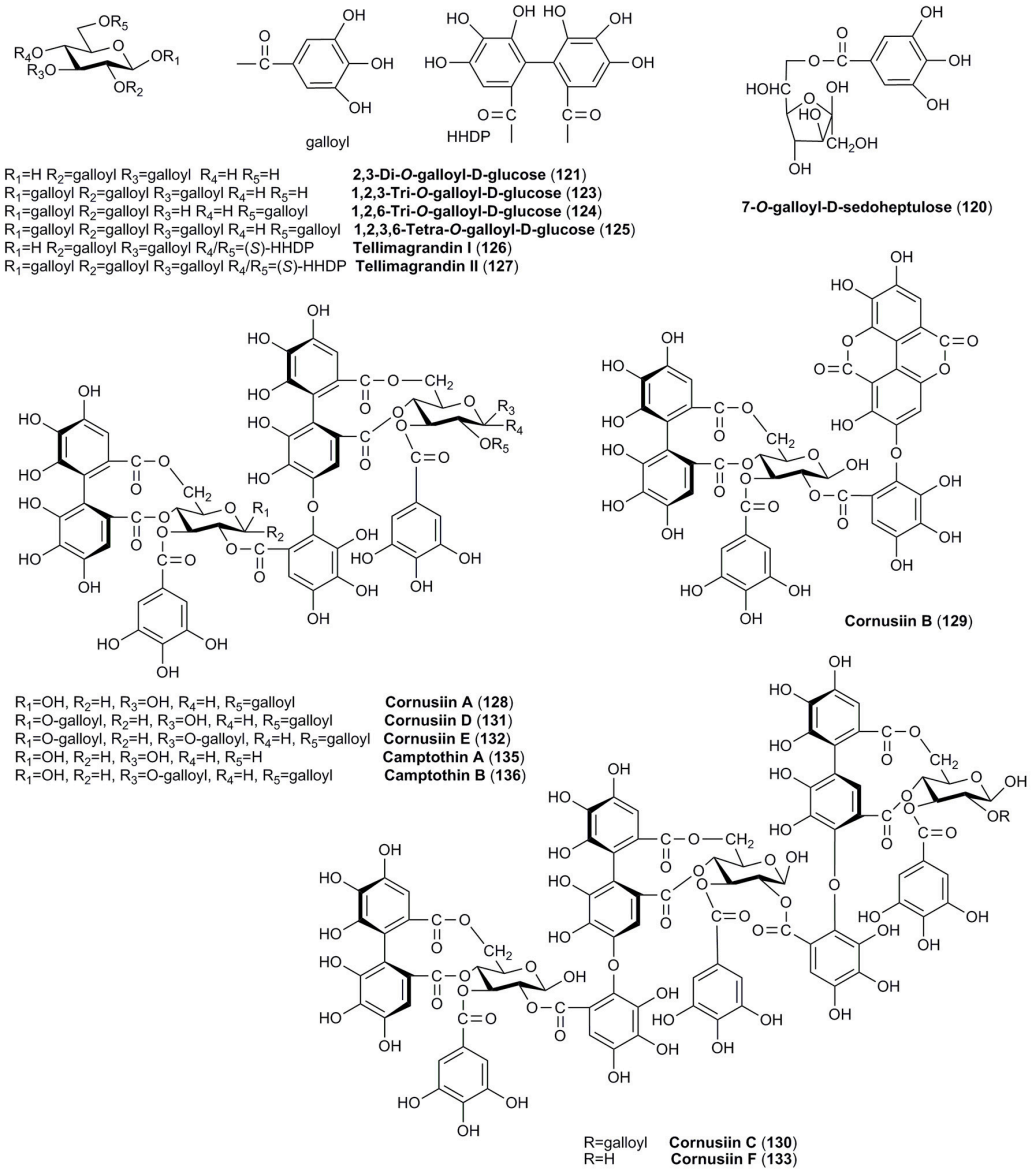
The chemical structures of tannins identified in fruits of *C. officinalis*.

**Figure 6:**
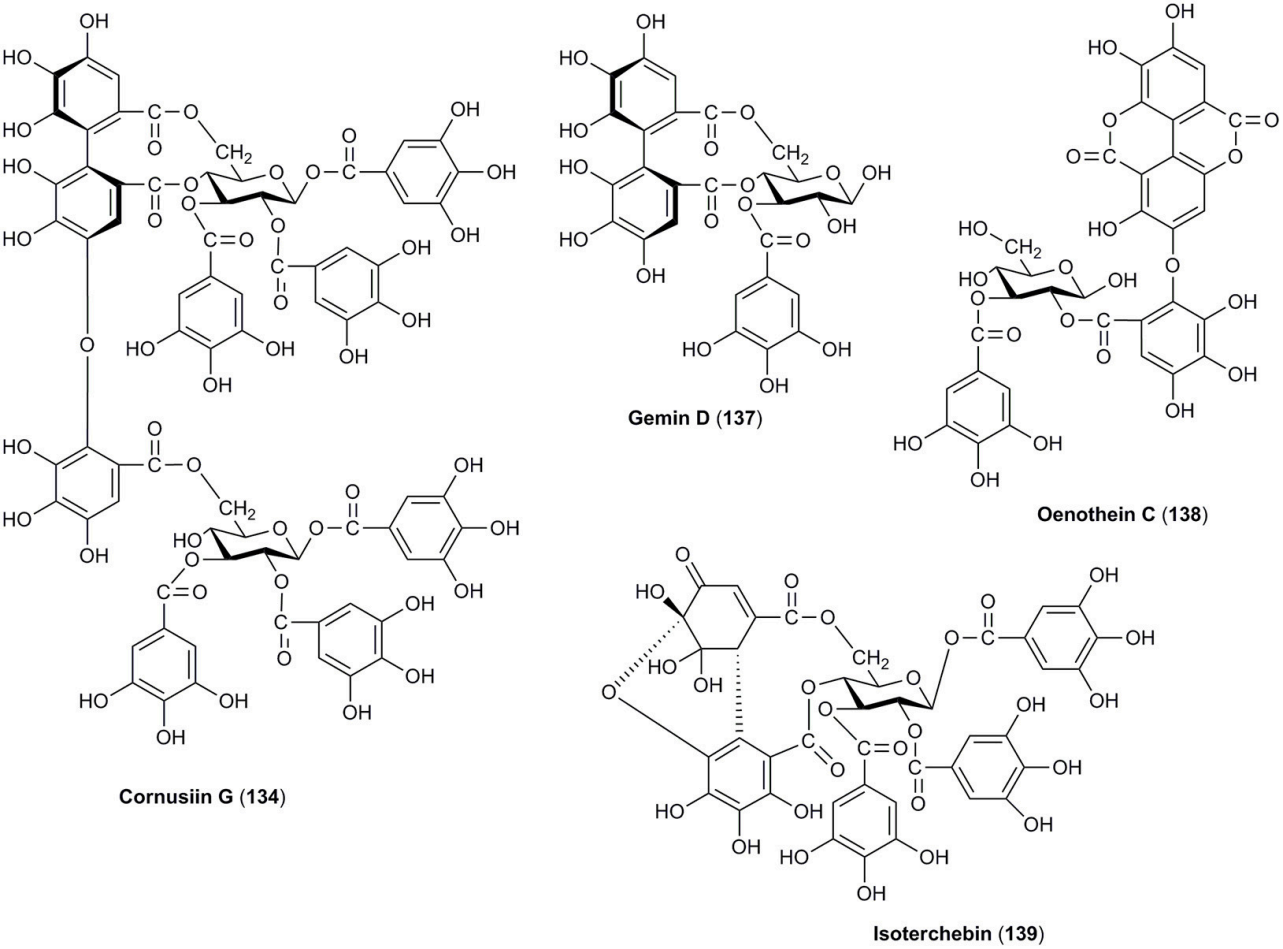
The chemical structures of tannins (continued) identified in fruits of *C. officinalis*.

Similarly, tannins are attributed only to the fruits of *C. officinalis* and have been widely described. Therefore, tannins (Figures 5, 6) in the fruits of *C. officinalis* constitute the most important difference between both plant materials. Despite the mean content of tannins in fruits of *C. mas* was established as 0.34% (Gunduz et al., 2013), the detailed reports on the identification of tannins in the fruits of *C. mas* have been limited in contrary to *C. officinalis*. The Asiatic dogwood is particularly considered a rich source of hydrolyzable tannins (Okuda et al., 1981, 1984). Twenty tannins (**120–139**), including ellagitannins and gallotannins, have been described in *C. officinalis*. The derivatives of galloylglucose (**121–125**), particularly digalloylglucose, have been identified to date. Tellimagrandin I (**126**) and II (**127**) as well as gemin D (**137**) and oenothein C (**138**) are representative of ellagitannins (Okuda et al., 1981, 1984; Lee et al., 1989; Cao et al., 2012; Bhakta et al., 2017). Most of unique dimeric and trimeric hydrolyzable tannins named cornusiins A–G (**128–134**) have been found only in the fruits of *C. officinalis* to date (Okuda et al., 1984; Hatano et al., 1989a,b, 1990). One of the bioactive constituents which should be mentioned due to the fact of potential biological activity is also 7-*O*-galloyl-D-sedoheptulose (**120**) (Yamabe et al., 2009; Yokozawa et al., 2010; Liu Z. et al., 2011; Park et al., 2015). It is worth to note that some tannins were detected in the fruits of cornelian cherry (Kucharska, 2012). However, their detailed identification was not conducted, and in fact the data concerning tannins in *C. mas* are not available.

#### Carotenoids and fatty acids of *C. mas*

Some lipophilic compounds such as carotenoids as well as fatty acids have been identified in the fruits of *C. mas* in contrast to *C. officinalis*. One report showed the determination of ten carotenoids (**140–149**) in the fruits of *C. mas* (Horváth et al., 2007), in addition to their quantitation in fresh and dried fruits (Rosu et al., 2011; Lofti et al., 2014). Carotenoids are believed to be regulators of lipid metabolism and antioxidants. Among carotenoids the most abundant in a diet is β-carotene (**147**), known singlet oxygen quencher and precursor for vitamin A (Grune et al., 2010). Despite limited data on this group of compounds in fruits of *C. mas*, their potential role in biological effectiveness of *C. mas* fruits should be considered. To the best of our knowledge there is no report on carotenoids in *C. officinalis*. Similarly, the available data on fatty acids in the fruits of Asiatic dogwood have been limited and the detailed analysis seems to be necessary. Fatty acids (**150–162**) such as linoleic acid (**152**), oleic acid (**155**) and palmitic acid (**156**) have been the most abundant in the fruits of *C. mas* (Krivoruchko, 2014).

#### Diversity of phenolic compounds in *C. mas* and *C. officinalis*

Nine quercetin derivatives (**67–75**) have been identified in the fruits of *C. mas* (Pawlowska et al., 2010). Simultaneously, kaempferol 3-*O*-β-D-galactoside (**77**), myricetin (**81**), and naringenin 7-*O*-methylether (**85**) have been detected in the fruits of *C. mas* along with 7,3′-dihydroxy-5,4′-dimethoxyflavanone (**86**) (Pawlowska et al., 2010; Rudrapaul et al., 2015). It is worth to note that 2*R*, 3*S*-*trans*-aromadendrin (**83**) and its 7-*O*-β-D-glucoside (**84**), which are not so widely distributed in plants compared to quercetin and kaempferol glycosides, were determined in the fruits of *C. mas* (Pawlowska et al., 2010; Rudrapaul et al., 2015). On the other hand, some glycosides, such as quercetin 3-*O*-β-D-glucuronide methyl ester (**74**), quercetin 3-*O*-β-D-(6″-*n*-butyl glucuronide) (**75**), kaempferol 3-*O*-β-D-rutinoside (**79**) as well as kaempferide **(80)** have been reported in fruits of *C. officinalis* according to the available literature (Lin et al., 2011; Xie et al., 2012; Ma et al., 2014).

Flavan-3-ols such as catechin (**88**) and epicatechin (**89**) have been identified and quantified in the fruits of *C. mas* (Capanoglu et al., 2011; Milenković-Andjelković et al., 2015). In the study of Milenković-Andjelković et al. (2015), catechin (**88**) was the predominant favan-3-ol in the fruit extracts of *C. mas* (Figure 3). Additionally, procyanidin B2 (**92**) was found only in the fruit extracts of *C. mas* (Radovanović et al., 2013; Milenković-Andjelković et al., 2015). The degree of polymerization in the fruits of cornelian cherry was assessed at the level of 63 total bound (epi)catechin units/end units (Capanoglu et al., 2011).

The mixture of benzoic acid (**98**) and cinnamic acid (**99**) derived phenolic acids, including ferulic acid (**100**), sinapic acid (**101**) as well as vanillic acid (**104**), was identified in the fruits of *C. mas* (Radovanović et al., 2013; Milenković-Andjelković et al., 2015; Rudrapaul et al., 2015; Cosmulescu et al., 2017). In addition, the esters formed between phenolic acids' particles, such as rosmarinic acid (**105**) and chlorogenic acid (**106**) have been found only in the fruits of cornelian cherry (Figure 4; Deng et al., 2013; Antolak et al., 2017; Popović et al., 2017; Bajić-Ljubičić et al., 2018). The unique derivatives of phenolic acids such as tachioside (**108**), caffeoyltartaric acid dimethyl ester (**110**), and caftaric acid monomethyl ester (**111**) are characteristic for Asiatic dogwood's fruits (Figure 4; Lin et al., 2011; Ma et al., 2014; Park et al., 2016).

#### Other constituents

Due to the fact of the usage of *C. mas* for a production of concentrates, jams, and even more often fruit juice and alcoholic beverages, rheological and physicochemical properties related to the quality of liquid foods have been reported (Güleryüz et al., 1998; Brindza, 2009; Kalyoncu et al., 2009; Karadeniz et al., 2009; Yilmaz et al., 2009; Hassanpour et al., 2012; Gastoł et al., 2013; Bilejić et al., 2015; Rad et al., 2016; Bozdogan, 2017). In particular, apart from titrable acidity, soluble solid content as well as total sugars and pectin content, vitamin C was often quantified in the fruits of *C. mas*. They were characterized by the high content of ascorbic acid ranging from 43.6 to 76.7 mg/100 g, or even reaching 112 mg/100 g in Turkish cultivars (Güleryüz et al., 1998; Çopur et al., 2003; Demir and Kalyoncu, 2003; Brindza, 2007, 2009; Yalcinkaya et al., 2009; Yilmaz et al., 2009; Rop et al., 2010; Dokoupil and Rezníček, 2012; Hassanpour et al., 2012; Bilejić et al., 2015; Kostecka et al., 2017). However, it was noted that cornelian cherry fruits from Greece were characterized by higher content of vitamin C than these ones from Poland (Kostecka et al., 2017). On the other hand, some physicochemical properties of cornel dogwood wine during its fermentation and storage were studied once (Zhang Q. -A. et al., 2013).

**Figure 7:**
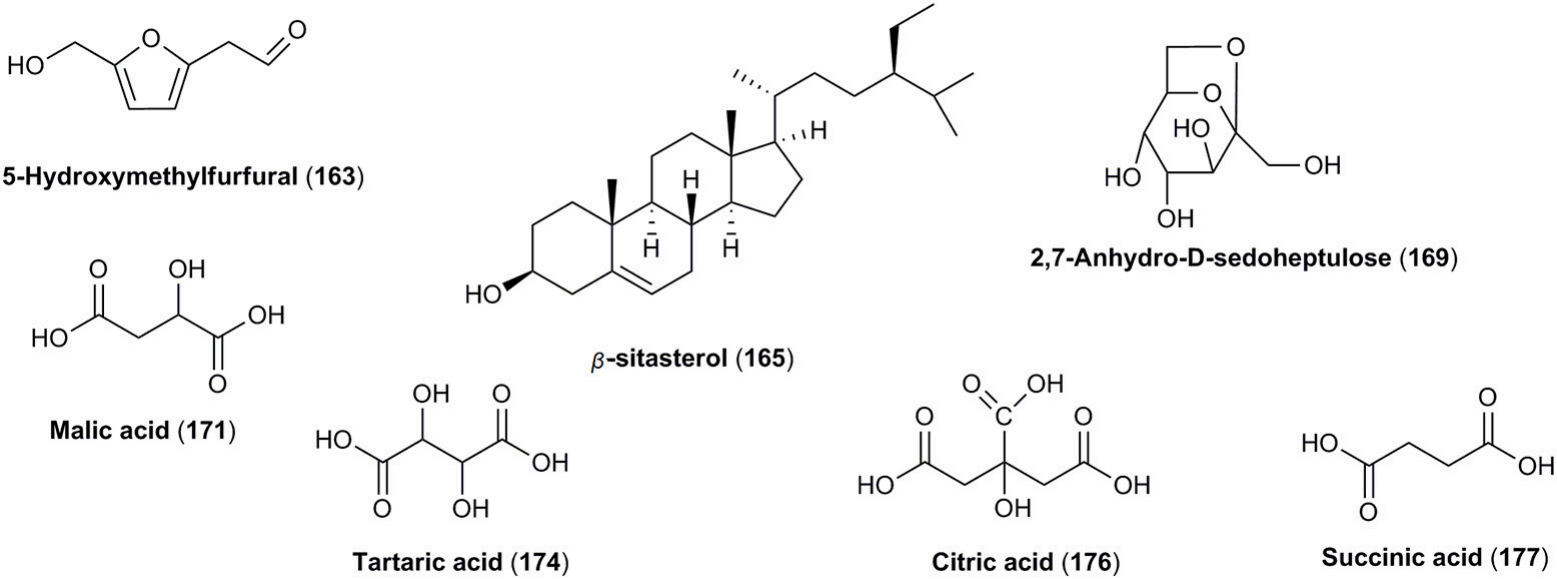
The chemical structures of other compounds identified in fruits of *C. mas* and/or *C. officinalis*.

Few reports considered the potential bioactivity of 5-hydroxymethylfurfural (**163**) isolated from fruits of *C. officinalis* (Figure 7; Du et al., 2008; Cao et al., 2011, 2012; Jiang et al., 2016). Additionally, steroid compounds including β-sitosterol (**165**) and daucosterol-6′-malate (**166**) have been identified only in fruits of *C. officinalis* (Figure 7; Lee et al., 1989; Lin et al., 2011; Xie et al., 2012).

## Similarities and differences of pharmacological effects or biological activities

### General

Taking into account different medical approaches in the traditional medicine in ancient Europe and Asia, in particular China, it is rather interesting that there are some similar indications reported for both species. They have been traditionally used to improve liver and kidney functions and are considered therapeutically usefull due to their antidiabetic and antimicrobial properties. Considering the phytochemical similarities between both species the comparable use of both species is scientifically reasonable. For this reason, the antidiabetic effect as well as renal and hepatic protective activities of both species were particularly considered in the reported studies. In addition, the positive effects on central nervous and cardiovascular systems of both species have been noted. The background of these effects seems to be the antioxidant activity as well as an amelioration of inflammatory processes through regulation of NF-κB and MAPK signaling pathways by constituents of extracts from fruits of both species. The isolated constituents of fruits, such as cornuside (**6**), loganin (**1**), ursolic acid (**112**), anthocyanins, and more specific for *C. officinalis* morroniside (**8**), and 7-*O*-galloyl-D-seduheptulose (**120**), demonstrated the biological impact. However, the pharmacological effect of other phytochemicals found in fruits of both species should not be excluded in the provided reports. Thus, the accurate qualitative and quantitative characterization of phytochemicals in extracts is required in the studies, and the beneficial role of complex phytochemical formulation providing the synergistic or additive effect should be underlined.

The antimicrobial and antitumor activities of both species seem to be mostly related to the presence of polyphenolic compounds in fruit extracts. However, these activities of both species do not seem to be well-established.

Table 2 demonstrates similarities and differences of the biological or pharmacological activities between the preparations from fruits of both species. The biologically or pharmacologically active groups of compounds or isolated constituents of fruit extracts were included in Table 2.

**Table 2:** Similarities and differences of the biological or pharmacological activities of fruits from *C. mas* and *C. officinalis*.

**Biological and pharmacological activities**	**Preparations or characterized natural products responsible for the activity of the fruits**	
	***Cornus mas^a^***	***Cornus officinalis^b^***
Antidiabetic activity	Hydroalcoholic extracts Dried fruits (powder) Anthocyanins Ursolic acid	Hydromethanolic extract Ethanolic extract Aqueous extracts Dried fruit powder 7-*O*-galloyl-D-sedoheptulose Loganin Morroniside Ursolic Acid Oleanolic acid
Antihyperlipidemic activity	Dried fruits (powder) Anthocyanins Loganic acid Ursolic acid	Not reported
Anti-inflammatory activity	Dried powder Polyphenols-rich extract Loganic acid	Aqueous extract Cornuside
Neuroprotective Anti-amnesic activity	Dried fruits (powder)	Methanolic extract Iridoid glycosides *p*-Coumaric acid Ursolic acid Gallic acid
Hepatoprotective activity	Hydromethanolic extract	Aqueous extract
Antioxidant activity	Dried fruits (powder) Ethanolic extract	Ethanolic extract Aqueous extract Iridoid glycosides Polysaccharides
Anticoagulant activity	Dried fruits (powder) Hydromethanolic extract	Not reported
Cardioprotective activity	Hydromethanolic extract Aqueous extract	Aqueous extract Cornuside
Kidneyprotective activity	Hydromethanolic extract	Total triterpene acids
Antitumor activity	Hydromethanolic extract Ethanolic extract Aqueous extract	Hydromethanolic extract Ethanolic extract Aqueous extract
Antimicrobial activity	Hydromethanolic extract Ethanolic extract Aqueous extract Dried fruit powder	Aqueous extract
Insecticidal activity	Not reported	Methanolic extract
Antiosteoporotic activity	Not reported	Aqueous extract
Immunomodulatory activity	Not reported	Aqueous extract
Ophtalmic activity	Anthocyanin-iridoid fraction Loganic acid	Not reported

a *Modified according to Dinda et al. (2016) and Hosseinpour-Jaghdani et al. (2017)*.

b *Modified according to Huang et al. (2018)*.

### Similar biological or pharmacological activities of *C. mas* and *C. officinalis*

#### Antidiabetic effect

Comparing the reported traditional therapeutic use of fruits from *C. mas* and *C. officinalis* it is obvious that the treatment of metabolic disorders played an important role. Based on different fundamentals of ancient medicine in China and Europe or western Asia and despite missing modern diagnostic tools for precise detection of diabetes, the physicians recognized definitely metabolic disease in the distant past and tried to treat their accompanying symptoms. Under this point of view the use of fruits from both species should be discussed in order to their potential usefulness in the treatment of metabolic diseases in different geographic regions. Due to the wide spectrum of natural compounds and modern investigations of their biological activity an antidiabetic effect of cornelian cherry seems to be proven. Based on some animal experiments investigating the effect of preparations made from *C. mas* fruits the blood glucose decreasing activity and enhancement of insulin sensitivity were demonstrated (Jayaprakasam et al., 2006; Mirbadalzadeh and Shirdel, 2012; Narimani-Rad et al., 2013; Asgary et al., 2014). The same findings were reported also for fruits of *C. officinalis* (Park et al., 2011; Gao et al., 2012; Liu et al., 2012; Han et al., 2014). Data from clinical studies evaluating the therapeutic effect of the herbal preparation are available only for *C. mas*. One study on the fruit extract showed its antidiabetic effect associated with the reduction of risk factors for secondary diseases (Soltani et al., 2015). Comparing the chemical constituents present in both species and their biochemical or pharmacological effects some similarities can be seen. In the extract of both species polyphenolic compounds (e.g., flavonoids, anthocyanins), which inhibit α-glucosidase activity combined with decreasing postprandial blood glucose level, are present (Park et al., 2011; Asgary et al., 2014; Shishehbor et al., 2016). Ursolic acid (**112**) also present in both species is capable to stimulate the glucose uptake through the phosphorylation of insulin receptor (Zhang et al., 2006). Specific antidiabetic effects were reported for 7-*O*-galloyl-D-seduheptulose (**120**) and iridoids [e.g., loganin (**1**), morroniside (**8**)] present in *C. officinalis* (Yamabe et al., 2009; Park et al., 2010, 2013; He et al., 2016) as well as for cyanidin 3-*O*-galactoside (**58**) and cyanidin 3-*O*-glucoside (**59**) present in *C. mas* (Jayaprakasam et al., 2005). On the other hand, loganin (**1**) and ursolic acid (**112**) were not able to affect an expression of phosphoenolpyruvate carboxykinase (PEPCK) in rat hepatoma H4IIE cell line on the contrary methanolic extract of *C. officinalis* fruit (50 μg/ml) which exerted insulin mimetic effect in this manner, and one of its fraction enhanced insulin secretion by rat insulin-secreting BRIN-BD11 cells in response to high glucose concentration (16.7 mM; Chen et al., 2008). Although there is only one published clinical study of the fruit extract from *C. mas* the antidiabetic effect seems to be proven (Soltani et al., 2015).

The prolonged high blood glucose concentration leads to progressive lesions in heart, kidney, blood vessels and other organs. Moreover, increased intracellular reducing sugars and their derivatives may participate in glycation and formation of advanced glycation endproducts (AGEs) which play an important role in further development of chronic diabetic complications, atherosclerosis and aging process. The phytochemicals of *C. officinalis* fruits such as gallotannins, particularly 7-*O*-galloyl-D-sedopheptulose (**120**), and morroniside (**8**) inhibited AGE formation or inhibited expression of receptors for AGEs (Yamabe et al., 2009; Lee et al., 2011; Park et al., 2012b, 2015; Lv et al., 2016). Due to the series of diabetes type II complications, the inhibition of AGE formation seems to be a promising target for therapeutic intervention supporting the AGE-related disorders.

#### Antioxidant activity

In addition to the studies of total phenolic, flavonoid, and anthocyanins content, antioxidant properties of the extracts from cornelian cherries were determined in a correlation with these phytochemicals. Apart from polyphenols, ascorbic acid and carotenoids in *C. mas* play a significant antioxidant role. Thus, both antioxidants content and antioxidant capacity of dogwoods fruits are factors determining the quality of cultivars and basic physicochemical properties. *In vitro* assays such as free radical scavenging with DPPH (2,2-diphenyl-1-picrylhydrazyl radical), ferric ion reducing antioxidant power (FRAP), total antioxidant capacity with ABTS^+^ (2,2-azinobis(3-ethylbenzothiazoline-6-sulfonic acid) radical cation) and oxygen radical absorbance capacity (ORAC) were the most frequently used methods in a wide range of studies on *C. mas* (Dragović-Uzelac et al., 2007; Pantelidis et al., 2007; Hamid et al., 2011; Celep et al., 2012; Popović et al., 2012; Gunduz et al., 2013; Pyrkosz-Biardzka et al., 2014; Stankovic et al., 2014; Milenković-Andjelković et al., 2015; Hosu et al., 2016; Moldovan et al., 2016) and *C. officinalis* (Lee et al., 2006; West et al., 2012a; Hwang et al., 2016). Moreover, the hydromethanolic extracts of some cornelian cherry fruit cultivars from Turkey demonstrated significant H_2_O_2_ scavenging activity (79.1%; Ersoy et al., 2011). Galactosides of cyanidin (**58**) and pelargonidin (**53**) detected in both species inhibited lipid peroxidation by 70.2 and 40.3% in an iron-catalyzed liposomal model, respectively (Seeram et al., 2002). On the other hand, the antioxidant properties, including protection of catalase and glutathione peroxidase, of *C. officinalis* fruits are also assigned to polysaccharides (Li et al., 2012; Wu et al., 2013) and ursolic acid (Yu et al., 2009).

#### Anti-inflammatory activity

Anti-inflammatory and analgesic activities of extracts are reported for both species. As mentioned before most of the results were obtained in cell culture or biochemical experiments. The beneficial effect of aqueous extract from fruits of *C. officinalis* and its iridoid constituent cornuside (**6**) on protein expression of COX-1 and COX-2 as well as PGE_2_ production in LPS-induced macrophages RAW 264.7 was shown (Sung et al., 2009; Choi et al., 2011). Similarly, ursolic acid (**112**), which was isolated from an ethanolic extract of *C. officinalis* inhibited NF-κB activation in LPS-stimulated peritoneal macrophages. Oral administration of ursolic acid (**112**) significantly inhibited 2,4,6-trinitrobenzenesulfonic acid (TNBS)-induced colon shortening and MPO activity in mice, and also suppressed TNBS-induced COX-2 and iNOS expression as well as NF-κB activation in colon tissues. Ursolic acid (**112**) may ameliorate colitis by regulating NF-κB and MAPK signaling pathways via the inhibition of LPS binding to toll-like receptors 4 (TLR4) on immune cells (Jang et al., 2014). Taking into consideration a wide range of ingredients and general mechanisms responsible for induction or prevention of inflammation, *in vitro* results fit to conclusions obtained from animal experiments and a clinical study (Sung et al., 2009; Sozanski et al., 2014, 2016; Moldovan et al., 2016). In particular, cornuside (**6**) was indicated as an immunomodulatory dogwood-derived compound in a model of sepsis in rats (Jiang et al., 2009). The link between chronic inflammation and cancer involves cytokines and mediators of inflammatory pathways. Plants contain numerous secondary metabolites shown to modulate inflammatory pathways. Studies have shown that these metabolites, among which some are used in traditional medicine, can reduce inflammation and carcinogenesis (Desai et al., 2018). Phenolic and other compounds present in the extracts of the dogwood fruits target various inflammation-related molecules and pathways associated with cancer (Choi et al., 2011). Polyphenols modulate important cellular signaling processes such as NF-κB (nuclear factor κ light-chain enhancer of activated B cells) activation, chromatin structure, glutathione biosynthesis, Nrf2 (nuclear redox factor) activation, direct ROS scavenging or antioxidant effect via glutathione peroxidase activity. Finally, they regulate inflammatory genes in macrophages and lung epithelial cells. Thus, recent data suggest that polyphenols can play a role of modifiers of signal transduction pathways to elicit their beneficial effects. However, to correlate this with beneficial effects in humans their metabolism and bioavailability need to be considered (Rahman et al., 2006).

#### Renal and hepatic protective activities

The metabolism of xenobiotics is often linked with the production of harmful radicals or metabolites damaging such organs like liver and kidney resulting in inflammation or carcinogenic transformation. As mentioned before natural products present in fruits of both species are able to inhibit both damage generated by radicals and inflammatory processes *in vitro*. Animal experiments performed with methanolic extracts of *C. mas* fruits are reported to show hepatoprotective activities against chemically induced hepatotoxicity after oral application. The dosage used in the investigations with rats ranging from 200 to 700 mg/kg body weight (Abbasi et al., 2014; Alavian et al., 2014; Saei et al., 2016). Similar reports concerning *C officinalis* are also published (Lee N. H. et al., 2012). The protective effect on kidneys is described for both species using comparable animal experimental settings (Yokozawa et al., 2008; Jiang et al., 2012; Abbasi et al., 2014; Es Haghi et al., 2014). In particular, the iridoids [loganin (**1**) and morroniside (**8**)] present mainly in the fruits of *C. officinalis* as well as 7-*O*-galloyl-D-sedoheptulose (**120**) are able to prevent renal damage in diabetic rats (Xu et al., 2006; Park et al., 2012a).

#### Antimicrobial effect

Antibacterial effects are also reported for extracts of the fruits from both species without remarkable activity (Mau et al., 2001; Krzyściak et al., 2011; Turker et al., 2012; Radovanović et al., 2013; Kyriakopoulos and Dinda, 2015; Milenković-Andjelković et al., 2015; Hosseinpour-Jaghdani et al., 2017). Due to the presence of a complex of different phenolic compounds in dogwood fruits the antimicrobial activity is not surprising. All phenolic compounds to a certain extent express this activity (Radulović et al., 2013). In addition, it is worth to note that some phytochemicals of plant materials may reduce the adhesion of bacteria (*Asaia* spp.) found in food products/beverages, as it was shown in the case of juices of cornelian cherry (Antolak et al., 2017). Thanks to this, the antiadhesive activity of fruit-derived constituents prevents the formation of biofilm on the solid surface, which can be potential source of product contamination. Thus, they are likely to protect both food and plant preparations used in the phytotherapy against loss of organoleptic quality manifested by turbidity and flock formation.

#### Effects on memory and brain functions

The phytotherapy of diseases or complaints related to the central nervous systems, including memory retention, has a long tradition in Asian and also in European medicine. The reported anti-amnestic and neuroprotective activities of both species seem to be based on similar constituents such as flavonoids and terpenoids which express antioxidant or more precisely radical scavenging effects (Dinda et al., 2016; Cooper and Ma, 2017; Huang et al., 2018). Animal experiments as well as biochemical investigations showed that the dried fruit powder and fruit extracts were able to increase level of physiological antioxidants such as glutathione and antioxidative enzymes, including superoxide dismutase or catalase, as well as to prevent cells and tissues against free radicals in this manner (Lee et al., 2006; Kim et al., 2011; Celep et al., 2012; Lee N. H. et al., 2012; Sozanski et al., 2014). In particular, morroniside (**8**) has been proved to protect human neuroblastoma cells against H_2_O_2_-induced stress, inhibit the apoptosis and accumulation of intracellular Ca^2+^ as well as restore the mitochondrial potential in SH-SY5Y cell line (Wang W. et al., 2008; Wang et al., 2009). The results of *in vitro* studies in cellular models were confirmed in cerebral ischemia model in rats. Morroniside (**8**) at the concentration range from 30 to 270 mg/kg body weight significantly reduced infarct volume, alleviated neurological impairment caused by ischemia/reperfusion-induced brain injury as well as enhanced progenitor cell proliferation (Wang et al., 2010; Sun et al., 2014a,b). Some special reports indicate the improvement of neurological functions in rats after feeding with iridoid glycosides from *C. officinalis* (Yao et al., 2009). Additionally, in the experimental autoimmune encephalomyelitis in animal models the administration of cornel iridoid glycoside significantly attenuated the symptoms of multiple sclerosis via an increased expression of brain-derived neurotrophic factor (BDNF) and nerve growth factor (NGF) as well as inhibition of JAK/STAT1 and JAK/STAT3 phosphorylation and reduction of proinflammatory mediators, such as TNF-α, IL-1β, IFN-γ, IL-17 (Yin et al., 2014; Qu et al., 2016). Iridoids of *C. officinalis* also protect glutamate-injured hippocampal cells (Jeong et al., 2012). A treatment of rats with a polyphenolic-rich dried fruits of *C. mas* resulted in increased activity of catalase in brain tissue, while in plasma it caused the opposite effect. On the other hand, the increased activity of paraoxonase-1 (PON1) was observed both in rats brain and plasma what potentiate a protective effect of PON1 with regard to the LDL and prevention of oxidative stress. Polyphenols and ascorbic acid of *C. mas* fruits were linked with this effect. Therefore, both neuroprotective and cardioprotective effects were assumed (Francik et al., 2014).

On the other hand, cornuside (**6**) as a representative for secoiridoid glycosides turned out to be an inhibitor of β-site amyloid precursor protein cleaving enzyme 1 (BACE1; IC_50_ = 55.8 μg/ml) but was a weak inhibitor of acetylocholinesterase (IC_50_ > 100 μg/ml) and butyrylocholinesterase (IC_50_ > 500 μg/ml). Other polyphenolic compounds isolated from fruits of *C. officinalis* such as tellimagrandin I (**126**), tellimagrandin II (**127**) and isoterchebin (**139**) were significant inhibitors of all enzymes engaged in the progression of neurodegeneration in Alzheimer's disease (Bhakta et al., 2017). Morroniside (**8**) was found to inhibit tau hyperphosphorylation, another Alzheimer's disease marker, via protein phosphatase 2A activation in human neuroblastoma SK-N-SH cells (Yang et al., 2016). The downregulation of mRNA expression of STIM1 and the inhibition of extracellular Ca^2+^ influx in PC12 cell line by an aqueous extract from fruits of *C. officinalis* were proposed as the potential inducing mechanisms of neurite generation and differentiation (Wang et al., 2015).

#### Cardioprotective activity

In close relation to neuroprotection linked with antioxidant properties, antihyperlipidemic and cardioprotective activity of both herbal preparations were demonstrated by *in vitro* as well as *in vivo* experiments. As mentioned before these protective effects seem to be based on the same chemical constituents. In animal studies for both plant materials evidence was shown for antihypertensive effects, decreasing blood pressure and reduction of circulating cholesterol concentration (Park et al., 2009; Rafieian-Kopaei et al., 2011; Asgary et al., 2014; Sozanski et al., 2014, 2016; Hosseinpour et al., 2017). The ethanolic extract of *C. officinalis* fruits has significantly inhibited adipogenesis (87.1%) in 3T3-L1 preadipocytes (Roh and Jung, 2012; Kang et al., 2014). In addition, it is believed that the metabolic diseases potentially correlate with endocrine hormones due to considerable role of cortisol in cholesterol metabolism (Fraser et al., 1999). One report demonstrated reduction of cortisol concentration in plasma of hamsters after supplementation of *C. mas* fruits (Lofti et al., 2014). In relation to lipid metabolism, the ability of morroniside (**8**) and 7-*O*-galloyl-D-sedoheptulose (**120**) to decrease the expression of proteins associated with lipid homeostasis SREBP-1 and SREBP-2 (sterol regulatory element binding proteins) in kidneys of mice (*db/db*) and rats has been shown (Yokozawa et al., 2009; Park et al., 2010). The direct cardioprotective role of cornuside present in both species has been established. The decrease of infarct volume after myocardial ischemia/reperfusion injury in rats treated with cornuside (**6**) has been assigned to the reduction of polymorphonuclear leukocytes infiltration, myeloperoxidase (MPO) activity and malondialdehyde (MDA) production in myocardial tissue as well as lower serum-levels of pro-inflammatory mediators (Jiang et al., 2011). Furthermore, this compound dilated vascular smooth muscles through endothelium-dependent NO/cGMP signaling pathway (Kang et al., 2007). Unfortunately, no convincing human studies addressing these biological activities have been published so far. Although there is one published clinical study of a fruit extract from *C. mas* demonstrating its antidiabetic effect associated with the decreasing risk factors of cardiovascular diseases (Soltani et al., 2015). These results revealed a trend in another clinical trial toward amelioration of lipid profile and vascular inflammation following addition of *C. mas* extracts to the daily diet of dyslipidemic children and adolescents but these findings need to be verified by larger scale trials (Asgary et al., 2013). However, human studies with extracts of *C. officinalis* fruits have not been published so far. Discussing these effects with respect to the ethnopharmacological reports regarding the traditional use of both species in the past some evidence for beneficial effects on heart and circulation system may exist. This concerns especially the traditional use of *C. mas* to treat disorders or complaints related to aging processes like diabetes, ischemia or cardiovascular diseases (Dinda et al., 2016; Hosseinpour-Jaghdani et al., 2017). Due to the use of *C. officinalis* in TCM as a component of complex drug mixtures (Chen et al., 2008; Liu et al., 2013; Dai et al., 2016), a clear assignment to effects on heart and circulation system is rather difficult. However, in preparations of TCM used for treatment of diseases often related to aging processes the fruits of *C. officinalis* are abundant constituents (Chinese Pharmacopoeia, 2015).

#### Antitumor activity

Despite the reports of antitumor activity of *C. mas* and *C. officinalis* fruits, the data do not provide any evidence that the fruits of both species show any real antitumor activity. All results from the literature refer to cytotoxic activities in different tumor cell lines (A549, MCF-7, SKOV3, PC-3, HepG2, U937) but not to animal experiments or human studies (Chang et al., 2004; Yousefi et al., 2015). Even though a cytotoxic effect on tumor cells is observed, the antitumor activity is not fully addressed and cannot be interpreted. This kind of over-interpretation of *in vitro* studies occurs very often but has no pharmacological relevance. All polyphenolic compounds, including the most abundant constituents of dogwood fruit extracts, bind to cells in culture leading to the induction of the biological effect. Consequently, the cytotoxicity or inhibition of proliferation associated with changes in signaling pathways occur. Only a clear selectivity in cytotoxicity between tumor and non-tumor cells corresponding with appropriate animal *in vivo* experiments opens up the possibility for the statement of antitumor activity. The reported antioxidant activity for both species (Dinda et al., 2016; Hosseinpour-Jaghdani et al., 2017; Huang et al., 2018) might be a hint for protective effects against tumor-inducing free radicals but for both species only *in vitro* models have been used to demonstrate such effects (Chang et al., 2004; Chu et al., 2009; Turker et al., 2012; Yousefi et al., 2015). In particular, the flavonoids are regarded as safe and easily obtainable. For this reason, they are considered ideal candidates for cancer chemoprevention or associated agents in clinical treatment (Sak, 2014).

## Unique biological and pharmacological activities of *C. mas* and *C. officinalis*

### General

The unique anticoagulant and ophthalmic effects of dried fruits and extracts or anthocyanin-iridoid fractions from fruits of *C. mas* were assessed in the available reports. On the other hand, the antiosteoporotic, immunomodulatory and insecticidal properties of *C. officinalis* fruits, their extracts or constituents, such as morroniside (**8**), polysaccharides or 5-hydroxymethylfurfural (**163**), and dimethyl malate (**173**) were only found. Moreover, there is no available report on the antihyperlipidemic activity of *C. officinalis*. The circumstances of needs in the traditional eastern or western medicine requiring scientific confirmation are a pivotal factor determining scientific research. For this reason, these differences between *C. mas* and *C. officinalis* might arise from the lack of dedicated studies or even from different cultural needs resulting from the geographical distribution of both species.

### *Cornus mas* L.

#### Anticoagulant effect

Historical reports about an anticoagulant effect of fruits from *C. mas* do not exist. According to the interpretation of diseases of the circulation system, their etiology was rather assumed in disturbances of the digestion tract based on the theory of the four humors. The knowledge of functions and pathological significance of platelets or the blood coagulation system is the result of research in the twentieth century. For this reason, single reports about pharmacological effects of traditionally used herbal remedies on blood coagulation or platelet function are rather remarkable. Thus, it is worth to note that the dried fruit powder and hydromethanolic extracts of the fruits of *C. mas* showed an anticoagulant effect in animal experiments. In hypercholesterolemic rabbits the cornelian cherry powder reduced the plasma fibrinogen level after feeding over 60 days. The results indicated that consumption of this dried plant material is likely to be beneficial in atherosclerotic patients due to its reducing effects on fibrinogen (Asgary et al., 2010; Rafieian-Kopaei et al., 2011). A significant decrease in the platelet distribution width (PDW) was found in male rats treated with 50, 200, and 400 mg/kg body weight of a hydromethanolic extract over 3 weeks. The changes in the platelet-related parameters suggested platelet activity inhibition without bone marrow suppression in the treated groups in comparison with the control groups. Regarding to the secondary products present in the drug, it is evidence that antioxidants reduce the platelet activity through stimulation of prostacyclin synthesis and scavenging the synthesis-inhibiting peroxides. Recent studies have shown that polyphenols such as quercetin (**66**) and catechin (**88**), stop NADPH oxidase enzyme (present in platelets). Therefore, inhibiting the production of superoxide anion (·${\text{O}}_{2}^{-}$) and increasing the biological power of NO consequently regulate glycoprotein pine receptors (GPIIb-IIIa) on platelet membrane surface, that in turn inhibits platelets' activation and their adherence during inflammation and thrombosis processes. Decreasing effect of the fruit extract on platelet activity might classify it as an alternative for antiplatelet therapy in cardiovascular diseases (Abdollahi et al., 2014). Unfortunately, clinical studies investigating this aspect of activity have not been published to date. Despite some similarities in the phytochemical composition regarding the phenolic compounds between fruits from *C. mas* and *C. officinalis* anticoagulant effects of *C. officinalis* preparations are not reported in the traditional Chinese medicine. According to the theoretical fundamentals of Chinese medicine the blood coagulation system did not play any role in TCM. Nevertheless, a biochemical study also proposed an antiplatelet activity of this drug based on malic acid (**171**), citric acid (**176**), and succinic acid (**177**) identified in the fruits of *C. officinalis* (Zhang Q. -A. et al., 2013).

#### Ophthalmic activity

It is believed that anthocyanins as well as other antioxidants may improve the visual functions and the visual field of patients with normal-tension glaucoma via neuroprotective effect, inhibition of lipid peroxidation and improvement of vascular blood flow into the optic nerve. The progressive optic nerve damage accompanied by morphological changes, loss of the field of vision and even increased intraocular pressure are the symptoms of so called glaucoma. An interesting study on the effect of an anthocyanin-iridoid fraction derived from fruits of *C. mas* on intraocular pressure was conducted in rabbits. Intraocular pressure was decreased by 19 and 25% after 2–3 h intraconjunctival administration of the fraction or loganic acid (**3**) solution (0.7%), respectively (Szumny et al., 2015). This activity of cornelian cherries polyphenols is likely to provide beneficial effects on vascular flow in diabetic and hypertensive retinopathy.

### *Cornus officinalis* Sieb. et Zucc.

#### Antiosteoporotic activity

Osteoporosis as a chronic disease is closely related with aging processes in the elderly. Chinese herbal medicines have been used to treat this disease for a long time and among the preparations listed in Chinese Pharmacopoeia fruits of *C. officinalis* are also included (Huang et al., 2018). Based on the modern findings in pathogenesis and treatment of osteoporosis a variety of results from biochemical and cell biological experiments are published which support the idea that natural products present in the plant preparation play a beneficial role.

Extract of *C. officinalis* significantly inhibits RANKL-mediated osteoclast differentiation in a dose-dependent manner and RANKL-induced phosphorylation of p38 and c-JUN N-terminal kinase. In the same way, the protein expression of c-Fos and NFATc1 is also suppressed as well as the RANKL-induced degradation of IκB kinase (Kim J. Y. et al., 2012). Morroniside (**8**), one the main constituent of extracts from *C. officinalis* fruits, has been studied on osteoarthritis in human chondrocytes. This compound increased the levels of type II collagen, and aggrecan as well as improved the level of proteoglycans in cartilage matrix. In this manner, it supported the survival of chondrocytes with articular cartilage thickness (Cheng et al., 2015). Summarizing, the extracts of *C. officinalis* or individual compounds inhibit osteoclast differentiation not only in *in vitro* cellular models but also in osteoporosis animal models (Xia et al., 2014). Detailed animal studies or clinical trials are not published.

#### Immunomodulatory activity

The pivotal characteristics of immunomodulators in the maintenance of the human well-being and health cannot be overlooked. The sources of these medicinal remedies are part of long traditions in different regions of the world, such as traditional Chinese medicine, which have been developed through empirical experience. Traditional medicine employs a holistic approach to the prevention of disease and traditional herbal medicines are a source of components with therapeutic value (Tiwari et al., 2018). Many studies have been conducted also on preparations of *C. officinalis* and have revealed constituents that influence the innate immune system through the stimulation of macrophages and lymphocytes, and modulation of the cytokine profile, which leads to a reduction of the incidence of infections or allergic diseases.

In addition, allergic asthma is a chronic inflammatory disease of the airways, demonstrated by symptoms such as wheezing, coughing, breathlessness, and airways inflammation. Investigations in a mouse model of allergic asthma suggest that the therapeutic effects of *Corni fructus* are mediated by reduced production of Th2 cytokines (IL-5), eotaxin, and specific IgE as well as a reduced eosinophil infiltration (Kim S. H. et al., 2012). These findings are supported by animal experiments with polysaccharides from crude and processed *C. officinalis* fruits which have an enhanced effect on non-specific immunity, specific humoral immunity and specific cellular immunity in immuno-depressed mice (Du et al., 2008).

Along with the anti-inflammatory effects mentioned before, these results provide some evidence that the fruits of *C. officinalis* show immune-protective activity. Nevertheless, the evidence by human studies is still missing.

#### Insecticidal activity

An interesting biological activity is the insecticidal effect of 5-hydroxymethylfurfural (**163**) and dimethyl malate (**173**) determined in a bioassay-guided fractionation of methanolic extract from the fruits of *C. officinalis* against larvae of *D. melanogaster*. Acute toxicity against adults of *D. melanogaster* was determined showing LD50 value of 34.0 and 21.5 μg/adult for 5-hydroxymethylfurfural (**168**) and dimethyl malate (**173**), respectively (Miyazawa et al., 2003). Such investigations were reported rather infrequently. Nevertheless, they are important with respect to protect health and food quality in the past and presently. The use of such experiences could help to develop ecological strategies for modern food storage.

## Conclusion

The utilization of plant resources for nutrition and medicinal use in the past belongs to the process of civilization. The investigation of such remarkable fruits from both species is therefore not surprising. According to the different ideas regarding the origin of diseases and methods for therapeutic treatment, the different use of fruits from *C. mas* und *C. officinalis* in the West and the East could be explained. Despite all these differences it is rather surprising that such a level of consistency is evident. The chemical composition of *C. mas* and *C. officinalis* seems to be well-established. However, the availability of missing information on some groups of compounds, such as fatty acids, carotenoids and ascorbic acid in the fruits of *C. officinalis*, is still needed. Nevertheless, fruits of both species are rich in iridoids, anthocyanins, and flavonoids. The main difference in phytochemicals concern tannins, which were found only in *C. officinalis* and may potentially determine the biological activity in a significant manner. However, the role of tannins, except for 7-*O*-galloyl-D-sedoheptulose, was not widely considered in the biological activity of *C. officinalis* fruits. Taking into consideration the close phylogenetic relationship of both species the question about tannins in fruits of *C. mas* is open. On the other hand, tannins do not rather occur in the iridoid-rich plant materials but this hypothesis requires detailed elucidation in this case. Nevertheless, the transfer of medicinal experiences from the West to the East or *vice versa* cannot be excluded but is not documented in the literature for this two species. The explanation for this consistency seems to be rather the result of testing and empirical observation. Analyzing the ethnopharmacological data available under modern scientific aspects and correlating the phytochemical composition of both species, the results show that nearly the same ingredients are also attributed for the same activity or presumed therapeutic use. However, some data concerning the phytochemical composition as well as pharmacological activity of, particularly *C. officinalis*, are still missed or limited due to their availability in Chinese. Thus, only dedicated comparative investigations of *C. mas* and *C. officinalis* fruit preparations could really provide evidence of similarities or differences in the field of their phytochemical composition and biological or pharmacological properties. Last but not least is the assignation of extracts constituents potentially responsible for the specific activity. It means that the accordance between ethnopharmacological use of both plant materials should be based on the well-established chemical composition as well as simultaneous *in vitro* and *in vivo* studies, which may facilitate the identification of active constituents. Only these studies could validate careful observations of the users or physicians in the past.

## Author contributions

MC carried out a literature review in the field of phytochemistry and biological activity. MM designed the draft of the manuscript and carried out a literature review in the field of pharmacological activity. Both authors wrote the manuscript, revised and approved the final version of the manuscript.

### Conflict of interest statement

The authors declare that the research was conducted in the absence of any commercial or financial relationships that could be construed as a potential conflict of interest.
